# An emission-capacitated vehicle routing model for sustainable urban waste collection using hybrid guided local search

**DOI:** 10.1038/s41598-026-38829-5

**Published:** 2026-02-07

**Authors:** Qazi Salman Khalid, Shahid Maqsood, Jabir Mumtaz, Sheheryar Mohsin Qureshi

**Affiliations:** 1 Department of Industrial Engineering, University of Engineering and Technology, Peshawar, 25000 Pakistan; 2 College of Engineering and Computing, American University of Bahrain, Riffa, Bahrain; 3 College of Mechanical and Electrical Engineering, Wenzhou University, Wenzhou, China; 4 School of Computing, Engineering and Physical Sciences, University of the West of Scotland, Paisley, Scotland, UK

**Keywords:** Sustainable logistics, Waste collection optimization, Emission reduction, Hybrid metaheuristic, Vehicle routing problem, Energy science and technology, Engineering, Environmental sciences, Environmental social sciences, Mathematics and computing

## Abstract

Urban logistics services, such as municipal solid waste collection, play a crucial role in shaping cities’ sustainability. These services are significant contributors to fuel consumption, operational costs, and greenhouse gas emissions. Traditional vehicle routing models, such as the capacitated vehicle routing problem with time windows, typically focus on minimizing distance or cost, which indirectly impacts emissions. However, these models fail to address the growing need for sustainable and environmentally conscious logistics strategies. This study introduces the emission-capacitated vehicle routing problem with time windows (E-CVRPTW), a novel optimization formulation that explicitly integrates a load-dependent fuel consumption model and an emission objective. The formulation also incorporates fleet-level policy constraints, including a carbon budget and an emission-intensity ceiling, providing a more comprehensive approach to minimizing both operational costs and environmental impacts. To solve the E-CVRPTW, a hybrid guided local search (HGLS) approach is employed with additional embedded features: (i) a novel cheapest insertion first initialization to generate high-quality starting solutions; (ii) adaptive feature penalties to diversify the search, while controlled neighborhood switching between 2-opt and 3-opt moves ensures an optimal balance between intensification and diversification. These features help the proposed algorithm to achieve better optimization solutions. Moreover, a rigorous experimental protocol using the Solomon and Gehring-Homberger benchmark instances demonstrates that HGLS, with additional features, significantly improves fuel consumption and emission reductions compared to baseline heuristics. Furthermore, a real-world case study on municipal waste collection reveals that optimized routing plans reduce fuel consumption and CO_2_ emissions by 9–11% while lowering total costs by 8–9%. The optimized solutions also meet strict policy targets under constrained conditions, showcasing the potential of E-CVRPTW in real-world applications. A sensitivity analysis explores the trade-offs among fuel prices, carbon prices, and emission weights, providing valuable insights for decision-makers in urban service planning and sustainability-focused policy formulation.

**Supplementary Information:**

The online version contains supplementary material available at 10.1038/s41598-026-38829-5.

## Introduction

Global warming has emerged as one of the most pressing global concerns, with significant consequences for ecosystems and human well-being^1^. The transportation sector is a leading contributor, responsible for over 7.3 billion metric tons of CO_2_ emissions annually^2,3^. Within this sector, urban services such as municipal solid waste collection play a disproportionate role in local emissions and operating costs. However, traditional routing policies often focus on minimizing distance or monetary cost, only indirectly influencing fuel consumption and carbon output. Waste collection systems face growing sustainability challenges as global solid waste generation, recorded at 2.01 billion tons in 2016, is projected to rise to 3.40 billion tons by 2050 due to rapid urbanization and population growth^4^. Inefficient collection practices not only elevate economic costs but also accelerate environmental degradation. The waste collection vehicle routing problem (WCVRP) exemplifies this issue: suboptimal routing increases fuel consumption, lengthens travel times, and amplifies emissions, undermining broader sustainability targets^5^. Addressing these inefficiencies through advanced vehicle routing optimization has therefore become a cornerstone of sustainable waste logistics^6^.

Classical vehicle routing problem (VRP) models, while effective at reducing distance or operating costs, are limited in their ability to address the multifaceted constraints of modern logistics, including carbon emissions, time windows, and heterogeneous capacity constraints. Recent studies in green vehicle routing have introduced load-dependent fuel models and explicit emission objectives, demonstrating that environmental performance can be significantly improved when carbon considerations are embedded directly into optimization models^7^. We address this gap by formulating an emission-capacitated vehicle routing problem with time windows (E-CVRPTW). The model integrates load-dependent fuel consumption with explicit emission objectives and incorporates fleet-level constraints, such as carbon budgets and emission-intensity ceilings. Time-window constraints further align the model with operational realities where missed or late services incur penalties and service failures. Since the VRP and its green variants are NP-hard, optimal solutions cannot be guaranteed for large-scale problems. This has led to widespread reliance on heuristics and metaheuristics. However, traditional approaches often struggle with balancing exploration and exploitation in multi-objective, large-scale contexts^8^. To overcome these limitations, this study introduces a hybrid guided local search (HGLS) algorithm. The method adaptively switches between neighborhood operators: 2-opt for rapid improvements in simpler cases and 3-opt for deeper exploration in complex instances, while employing dynamic thresholding and penalty adaptation to ensure computational efficiency. This hybridization enables robust performance across varying problem complexities, ensuring that both operational efficiency and sustainability objectives are addressed.

Although the proposed problem is related to the green vehicle routing problem (GVRP), it is not equivalent to classical carbon-capacitated formulations. Most existing GVRP variants impose emission or fuel-consumption limits at the level of individual vehicles, routes, or arcs, or enforce a fixed upper bound on total emissions. In contrast, this study incorporates fleet-level environmental policy instruments, including a fleet-wide emission-intensity ceiling and a total fleet emission cap, which are enforced at the aggregated fleet level. The emission-intensity constraint couples routing decisions across all vehicles through a global distance-emission relationship and therefore cannot be decomposed into independent per-vehicle constraints. This structure reflects regulatory mechanisms applied at the fleet level and motivates the proposed formulation. Moreover, urban logistics accounts for a large share of local air pollutants and greenhouse gas emissions, yet classical VRPTW formulations minimize distance or cost and only indirectly influence emissions. We address this gap by formulating the E-CVRPTW, which embeds a load-dependent fuel-consumption model and explicit emission objectives while enforcing fleet-wide policy instruments, namely a total fleet emissions cap (*E*_*max*_) and a maximum allowable average emission intensity (η_*max*_, kg CO_2_ per kilometer).

i. A complete E-CVRPTW is formulated, integrating a load-dependent fuel-consumption model, explicit emission objectives, and fleet-level policy constraints, such as carbon budgets and emission-intensity ceilings.
ii. A hybrid guided local search (HGLS) metaheuristic is developed, initialized with a novel cheapest insertion with feasibility (CIF) heuristic that generates high-quality, constraint-compliant seed solutions, thereby improving convergence speed and solution quality.
iii. The HGLS framework introduces adaptive feature penalties and controlled neighborhood switching between 2-opt and 3-opt moves, improving the balance between intensification and diversification in large-scale problem instances.
iv. Extensive benchmark testing on Solomon and Gehring–Homberger instances, along with a real-world waste-collection case study and multi-scenario policy analysis, demonstrates the model’s effectiveness in reducing costs and emissions while providing actionable guidance for sustainability-oriented urban logistics.

The remainder of this paper is organized as follows. Section “Literature review” presents a comprehensive literature review, highlighting key contributions to the GVRP, its applications in municipal waste collection, and solution approaches, while identifying the research gaps that motivate this study. Section “Methodology” describes the research methodology, including the formulation of the E-CVRPTW and the design of the proposed HGLS algorithm. Section “Proposed solution approach” reports benchmark testing on widely used Solomon and Gehring-Homberger instances, validating the proposed method’s efficiency and scalability. Section “Experimental analysis” presents a real-world case study that applies the E-CVRPTW and HGLS framework to a municipal waste collection scenario under realistic operational and policy constraints. Section “Results” discusses the results of both benchmark and case study analyses, emphasizing improvements in cost, distance, and carbon emissions. Finally, “Conclusion” concludes the study with managerial implications, limitations, and directions for future research.

## Literature review

This section reviews prior work in four parts. First, the GVRP is discussed, followed by VRP applications in waste collection. Solution approaches are then considered, culminating in a synthesis of the research gaps that motivates this study.

### Green vehicle routing problem

The classical VRP primarily seeks to minimize distance or cost, indirectly influencing environmental outcomes. However, as sustainability concerns have deepened, researchers began reformulating the VRP to address emissions and fuel use directly. This gave rise to the GVRP, which integrates environmental performance indicators into the optimization process. Early research established foundational heuristics, such as Costa et al.^9^, who combined the Clarke-Wright heuristic with a GA and 3-opt moves to optimize CO_2_ emissions for diesel delivery vehicles, and Omidvar and Tavakkoli-Moghaddam^10^, who applied SA and GA to reduce fuel and energy consumption. These works demonstrated that routing optimization could deliver measurable sustainability gains without substantial cost penalties.

As computing power and data availability improved, the field transitioned toward richer formulations that capture real-world variability. For instance, Wang et al.^11^ proposed a GVRP incorporating time-varying speeds and cold-chain constraints, solved using adaptive large neighborhood search (ALNS), illustrating the integration of temporal dynamics into sustainable routing. Similarly, Wei et al.^12^ applied a multi-objective NSGA-II combined with A* search for demand-responsive shuttle services, balancing fuel savings with service reliability. Dewi and Utama^13^ hybridized whale optimization with tabu and local search to lower fuel consumption and emissions. Parallel to these efforts, Yavuz and Çapar^14^ examined mixed-fuel fleets, optimizing fuel-type adoption decisions using variable neighborhood search (VNS), while Soysal et al.^7^ applied dynamic programming heuristics to account for time-dependent fuel efficiency. These studies reflect a gradual shift from isolated, vehicle-level optimization to system-wide energy and emissions modeling, in which operational decisions explicitly influence environmental impact.

Recent GVRP research has also expanded toward multi-criteria optimization that jointly minimizes cost, emissions, and service reliability. Talouki et al.^8^ addressed perishable goods logistics using multi-objective formulations integrating green traffic and perishability constraints. Camacho-Vallejo et al.^15^, reviewed metaheuristics for bi-level optimization, emphasizing that cooperative logistics can balance profit and emissions. Liu et al.^16^ embedded carbon-trading mechanisms within cold-chain logistics, proving that policy-driven emissions constraints can reshape routing efficiency. Collectively, these studies illustrate a consistent trajectory from fuel-aware heuristics toward explicit emission-cap modeling and policy integration. The current study extends this trajectory by incorporating fleet-level constraints such as carbon budgets and emission-intensity ceilings within E-CVRPTW.

### Vehicle routing problem in waste collection

Municipal solid waste (MSW) collection provides one of the most practical and environmentally significant applications of GVRP concepts. Waste collection networks are inherently spatial, repetitive, and fuel-intensive, making them prime candidates for emission-conscious optimization. Traditionally, waste collection problems (WCPs) have been categorized into node routing problems (NRPs), where demand is at discrete points, and arc routing problems (ARPs), where demand is continuous along streets. The pioneering work of Beltrami and Bodin^17^ provided the first mathematical framework for waste-collection routing, defining constraints on vehicle capacity and service coverage. Subsequent advancements introduced time windows, multi-trip operations, and multi-compartment vehicles, progressively aligning waste collection with modern VRP formulations.

Contemporary reviews, such as those by Hess et al.^18^, highlight that NRPs and ARPs are now tailored for sustainability, emphasizing the integration of carbon and energy metrics. Grakova et al.^19^ exploited high-performance computing to improve municipal routing efficiency, while Henke et al.^20^ employed a multi-compartment CVRP to collect glass waste, achieving a 34.8% reduction in cost by optimizing compartment utilization. Tirkolaee et al.^21^ further extended these ideas to multi-trip VRPTW settings, minimizing transportation costs by 13.3% through an applied heuristic. Recent models, such as Liao et al.^22^, propose bi-objective optimization of cost and greenhouse gas emissions using learning-guided evolutionary algorithms, while Silva et al.^23^ developed an open-source CWCP solver that scales to large municipal networks.

These developments collectively show that waste collection routing has evolved from static distance minimization to dynamic, emission-explicit optimization, where the choice of fleet, scheduling, and policy constraints directly affects sustainability outcomes. However, many models still treat fuel and emissions as post-hoc estimates rather than embedded objectives. The present study addresses this shortfall by directly integrating a load-dependent fuel consumption model within the VRPTW framework, enabling real-time evaluation of emission trade-offs during optimization.

### Solution approaches

Given the NP-hard nature of waste routing problems, especially when emission, capacity, and time-window constraints are combined, researchers have progressively shifted from exact algorithms to hybrid metaheuristics. Early methods such as mixed-integer linear programming (MILP)^24^ and heuristic optimization offered theoretical precision but are computationally intractable for large urban systems^25^. Exact approaches based on route relaxation and pricing have been proposed for VRP variants, but their computational burden motivates efficient metaheuristics for operational-scale instances^26^. With the advancement of metaheuristic methods, many researchers are increasingly turning to them as a preferred approach for optimization^27–29^. Hybrid formulations have since bridged this gap: recent advances, such as the hybrid MILP-heuristic approach of Rekabi, Sazvar and Goodarzian^30^, demonstrate that combining exact formulations with metaheuristic refinements can yield practical computational gains for city-scale waste system integrated MILP with heuristic refinement to balance optimality and speed. Rizvanoglu et al.^31^ demonstrated up to 33% cost reduction in Turkish municipal systems using linear-programming-based optimization. To address computational challenges in larger and more complex problems, heuristic and metaheuristic methods are widely used. Glover^32^ defined the guiding principles of metaheuristics, emphasizing the dual balance of exploration and exploitation. These methods rely on two fundamental mechanisms: exploration and exploitation; to iteratively refine solutions and are often classified into two main categories: metaphor-based algorithms (e.g., GA^33^, PSO^34^, and ACO^35^ and non-metaphor-based algorithms (e.g., tabu search (TS) and VNS). Recent reviews, such as Li et al.^36^, reviewed recent adaptations of GA, PSO, ACO, and VNS for waste management, highlighting how sustainability metrics reshape classical VRP heuristics.

Empirical studies further validate this shift: Liao et al.^36^ used a learning-guided evolutionary algorithm to minimize costs and emissions simultaneously. In a similar direction, Elgarej et al.^37^ compared ACO and GA, showing that distributed, agent-based coordination can outperform traditional population-based search; and Hannan et al.^38^ optimized waste collection thresholds via PSO, achieving efficient collection cycles at 70–75% bin capacity. Similarly, Assaf and Saleh^39^ used GA to reduce total travel distance in Nablus City by 66%. Beyond single-algorithm designs, hybrid metaheuristics continue to expand their role. Silva^40^ investigated electrified waste fleets using hybridized heuristics that embed charging constraints into routing, showing significant reductions in carbon emissions compared with diesel-only fleets. Similarly, Shao et al.^41^ designed a waste collection synchronization mechanism using TS to maximize profitability. More recently, hybrid metaheuristics have emerged that combine global exploration with adaptive local refinement. These include Agent-Based Guided Local Search (GLS)^42^ which penalizes overused arcs to escape local minima, and the integration of GLS with evolutionary strategies to improve scalability and emission sensitivity. Such hybrids offer an effective middle ground, retaining the responsiveness of heuristics while maintaining the robustness of population-based search. The current research builds on this lineage through a HGLS that integrates a CFI seed with adaptive penalty adjustment and controlled 2-opt/3-opt neighborhood switching, ensuring both efficiency and sustainability in large-scale waste routing.

### Research summary

The literature on green vehicle routing has made notable progress by embedding emission objectives into routing models and applying metaheuristics across diverse transport sectors^6^, leveraging metaheuristics^9,15^. Within municipal solid waste management, recent studies^43^ have advanced capacity-constrained and time-window-based formulations, showing that optimized routing can reduce costs and improve service efficiency. However, explicit modeling of load-dependent fuel consumption, direct fuel–emission relationships, and fleet-level carbon constraints remains underexplored. Moreover, while metaheuristics have been widely applied, most approaches lack adaptive mechanisms to balance intensification and diversification in large-scale, multi-objective problems, and few employ rigorous experimental protocols with statistical validation. Real-world case studies are also limited in incorporating policy instruments such as carbon pricing or emission caps. This gap motivates our formulation of the E-CVRPTW, which couples a load-dependent fuel model with explicit emission objectives and optional fleet-level constraints. To solve large instances effectively, we propose an HGLS that integrates heuristic seeding, adaptive feature penalties, and controlled neighborhood switching, thereby ensuring robustness across benchmark and real-world waste-collection scenarios.

## Methodology

**Fig. 1:**
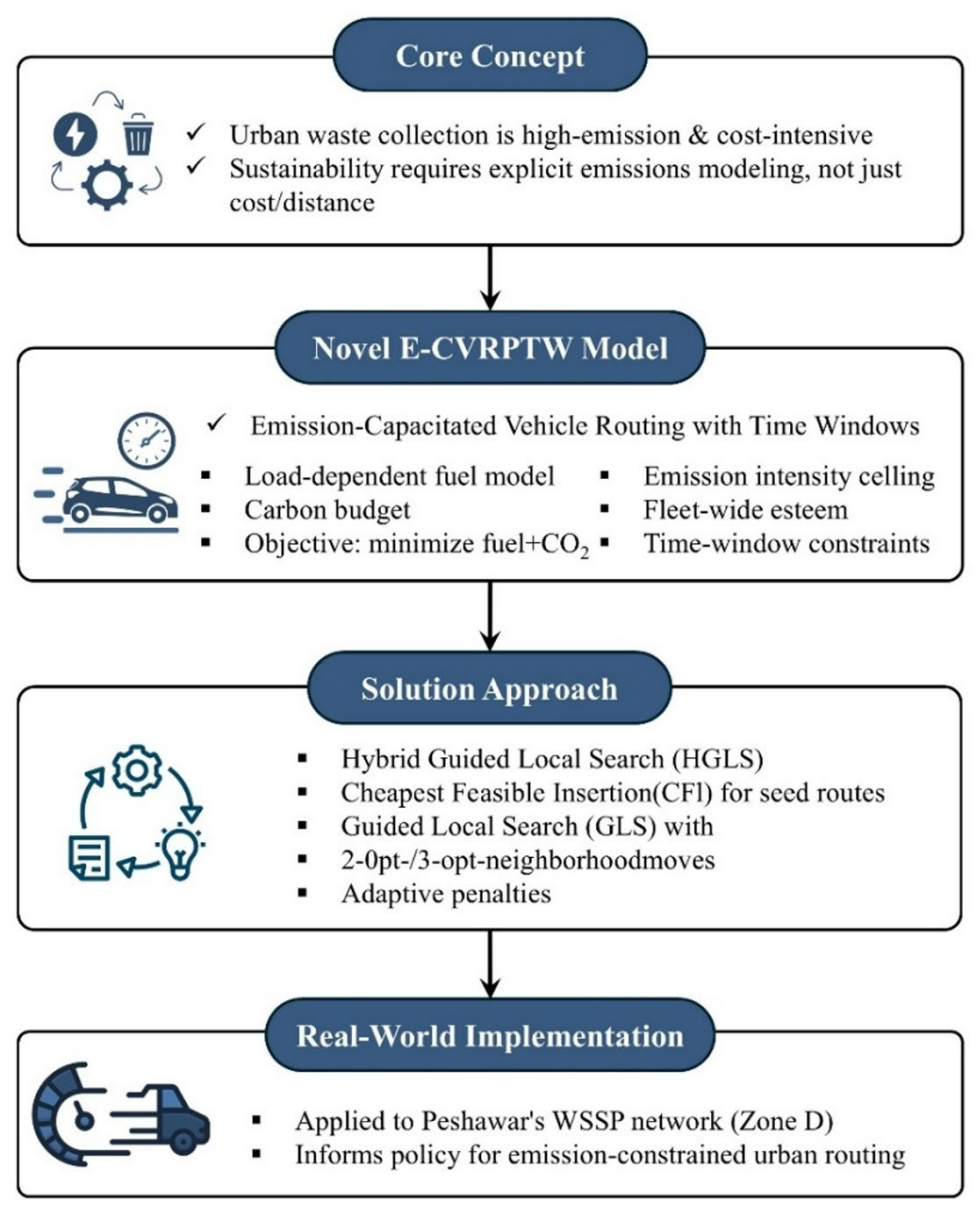
Overview of the proposed E-CVRPTW framework, highlighting the steps.

This research utilizes a three-stage methodology to tackle the research problem, focusing on model and algorithm development, validation, and practical implementation as shown in Fig. 1. This model incorporates environmental and operational parameters to align with sustainability goals. In the first stage, a mathematical model is developed to optimize waste collection routes, accounting for vehicle capacity, time constraints, and emission reduction. To optimize the model’s results, a hybrid algorithm is proposed to enhance its accuracy and efficiency. The second stage assesses the proposed algorithm through a thorough evaluation of benchmark problems. This step validates the algorithm’s efficiency and effectiveness by comparing its performance with standard datasets, demonstrating its ability to address complex routing challenges. In the third stage, the model is applied to a case study of Water and Sanitation Services Peshawar (WSSP). This implementation tailors the model to the organization’s waste-collection operations, demonstrating its potential to enhance operational efficiency and minimize emissions in practical settings.

### Model formulation

A city logistics distribution network consists of *m* distribution depots and *n* customers. Each vehicle has a maximum load capacity of *Q*. To comply with fuel consumption limits and driver workload regulations, the vehicle’s travel distance cannot exceed *L*. The goal is to optimize vehicle allocation and route design, while adhering to constraints on vehicle load and travel distance, to minimize logistics costs and the total distance traveled for distribution. To devise a feasible and scientifically sound distribution plan, the following assumptions are made in this study:

i. The number of vehicles available at each distribution depot is limited.
ii. Each vehicle departs from its designated depot, follows a distribution route to deliver goods to assigned customers, and returns to the same depot.
iii. A single vehicle can serve multiple customers; however, each customer is visited only once by one vehicle.
iv. All vehicles are subject to capacity and maximum travel distance constraints. The fleet is heterogeneous, with consistent characteristics such as vehicle speed, design parameters, and driver behavior.
v. The location and demand of each customer are known in advance, ensuring that link relationships and node distances are predetermined.
vi. The depot’s location is known.

To construct the model, the following parameters and variables are defined:

**Table:** 

**Sets:**	
$\:V$	$\:V=\left\{\mathrm{0,1},2,\dots\:,\:n\right\}$; Set of all nodes in the network, where 0 denotes depot and 1,2,3, …, n
$\:K$	$\:K=\:\left\{\mathrm{0,1},2,\dots\:,\:m\right\}$; Set of available vehicles in the fleet.
$\:A\:\:$	$\:A=\:\left\{(i,j)\right\}|\:i,\:j\:\in\:V,\:i\:\ne\:j$; Set of all directed arcs connecting distinct nodes *i* and *j*.
*R*	Set of all directed arcs connecting distinct nodes *i* and *j*.
**Parameters**	
*d*_*ij*_	Travel distance between node i and node j, measured over the actual road network.
*t*_*ij*_	Travel time between node i and node j
[*a*_*i*_,*b*_*i*_]	Service time window for customer i, where: a.i. is the earliest permissible start time and *bi* is the latest permissible start time.
*s*_*i*_	Service duration at node *i*, accounting for unloading/loading or waste collection time.
*q*_*i*_	Demand at customer *i*
*Q*	Maximum capacity of each vehicle (cubic meters)
*V*_*k*_	Volume capacity of vehicle *k*, used when demand is expressed in cubic meters.
*W*_*k*_	Weight capacity of vehicle *k*, used when demand is expressed in kilograms.
ρ_0_	Fuel consumption rate at empty load (liters/km)
ρ_1_	Fuel consumption rate at full load (liters/km)
$\:\gamma\:$	Carbon emission factor (kg CO_2_ per liter of fuel)
*p*_*f*_	Unit price of fuel (USD/liter)
*p*_*c*_	Unit price of carbon emissions (USD/kg CO_2_
*E*_*max*_	Maximum allowable total CO_2_ emissions for all vehicles combined (kg CO_2_)
η_*max*_	Maximum allowable average emission intensity, measured as kg CO_2_ per kilometer.
*F*_*max*_	Maximum allowable total fuel consumption for the fleet (liters).
*w*_*1*_,*w*_*2*_	Objective function weights for fuel consumption and emissions, respectively (*w*1 + *w*2 = 1)
*M*	A sufficiently large constant used in big-M formulations to deactivate constraints when an arc is not traversed.
$\:{B}^{{CO}_{2}}$	Maximum allowable fleet-level carbon emissions (carbon budget)
$\:\varPhi\:\left({L}_{i}^{k}\right)$	Load dependent fuel consumption rate function
**Decision variables**	
$\:{x}_{ij}^{k}\in\:\:\left\{\mathrm{0,1}\right\}$	1 if vehicle *k* travels directly from node *i* to node *j*, 0 otherwise
$\:{T}_{i}^{k}\ge\:0\:$	Start of service time for vehicle *k* at node *i*
$\:{W}_{i}^{k}\ge\:0$	Continuous variable denoting the waiting time experienced by vehicle *k* before starting service at node *i*. Waiting occurs when a vehicle arrives before the time window opens.
$\:{L}_{i}^{k}\ge\:0$	Load on vehicle *k* after servicing node *i*
$\:{F}_{ij}^{k}\ge\:0\:$	Fuel consumed by vehicle *k* on arc (*i*,*j*)
$\:{E}_{ij}^{k}\ge\:0$	Emissions produced by vehicle *k* on arc (*i*,*j*) (kg CO_2_)
$\:{U}_{i}^{k}\ge\:0\:$	Auxiliary continuous variable used in Miller–Tucker–Zemlin (MTZ) subtour elimination constraints. It typically represents the cumulative load or sequence position for vehicle *k* after visiting node *i*.

### Fuel consumption and emission model

Carbon dioxide emissions are directly proportional to fuel consumption, making fuel usage a reliable proxy for estimating emissions^44,45^. Fuel usage depends on both the load carried and the distance traveled. Following Suzuki’s load-dependent fuel consumption model^46^, we define *ρ*_0_ as the fuel consumption rate per kilometer for an empty vehicle and *ρ*_1_ as the rate for a fully loaded vehicle. Let $\:{L}_{i}^{k}$ denote the load on vehicle *k* immediately after departing from node *i*. The load-dependent fuel consumption rate per unit distance is given by:

$$\:\varPhi\:{(L}_{i}^{k})=\:{\rho\:}_{0}+\left({\rho\:}_{1}-\:{\rho\:}_{0}\right)\frac{{L}_{i}^{k}}{Q}$$

Since vehicle loads vary between nodes, fuel consumption also changes across arcs. The total fuel consumption for traveling from node *i* to node *j* using vehicle *k* is computed as:

$$\:{F}_{ij}^{k}=\left[{\rho\:}_{0}+\left({\rho\:}_{1}-\:{\rho\:}_{0}\right)\frac{{L}_{i}^{k}}{Q}\right].\:{d}_{ij}$$

The corresponding CO_2_ emissions for arc (*i*,*j*) are calculated as:

$$\:{E}_{ij}^{k}=\:\gamma\:.\:{F}_{ij}^{k}$$

This formulation ensures that both fuel consumption and emissions are explicitly linked to route structure and vehicle loading patterns.

### Mathematical model

The proposed emission-capacitated vehicle routing problem with time windows (E-CVRPTW) aims to minimize a weighted combination of total fuel consumption and total CO_2_ emissions across all vehicles and routes. This is expressed in Eq. (1), where *w*_1_ and *w*_2_ are user-defined weights such that *w*1 + *w*2 = 1, $\:{F}_{ij}^{k}$denotes the fuel consumed by vehicle *k* when traveling from node *i* to node *j*, and $\:{E}_{ij}^{k}$ represents the corresponding CO_2_ emissions. Given that emissions are proportional to fuel consumption via the emission factor *γ*, the objective can equivalently be expressed as a single weighted fuel term, [*w*1 + *w*2 *γ*] $\:{F}_{ij}^{k}$, without loss of generality. This formulation allows decision-makers to emphasize cost, environmental impact, or a balance of the two by adjusting the weights.

1 $$\:\mathrm{min}Z=\sum\:_{k\in\:K}\sum\:_{\left(i,j\right)\in\:A}({w}_{1}{F}_{ij}^{k}+\:{w}_{2}{E}_{ij}^{k}){x}_{ij}^{k}$$

This objective explicitly accounts for both fuel use and emissions, allowing decision-makers to prioritize cost, environmental impact, or a balance of the two by adjusting *w*1 and *w*2. The first set of constraints governs routing feasibility. Equation (2) ensures that each customer node is visited exactly once by exactly one vehicle, thereby enforcing complete service coverage. Equation (3) stipulate that each vehicle departs from the depot exactly once at the start of its route and returns to the depot exactly once at the end of its route. Equation (4) enforces flow conservation, requiring that the number of arrivals at a customer equals the number of departures for that customer in a given vehicle route.

2 $$\:\sum\:_{k\in\:K}\sum\:_{j\in\:V\backslash\:\left\{i\right\}}{x}_{ij}^{k}=1,\:\:\:\:\:\:\:\:\:\:\:\:\:\:\:\:\:\:\:\:\:\:\:\:\:\forall\:i\in\:V\setminus\:\left\{0\right\}$$

3 $$\begin{aligned}\:\sum\:_{j\ne\:0}{x}_{0j}^{k}\le\:1,\:\:\:\:\:\:\:\:\:\:\:\:\:\:\:\:\:\:\:\:\:\:\:\:\:\:\:\:\:\:\:\:\:\:\:\:\:\:\:\forall\:k\in\:K\\\:\sum\:_{i\ne\:0}{x}_{i0}^{k}\le\:1,\:\:\:\:\:\:\:\:\:\:\:\:\:\:\:\:\:\:\:\:\:\:\:\:\:\:\:\:\:\:\:\:\:\:\:\:\:\:\:\:\forall\:k\in\:K\end{aligned}$$

4 $$\:\sum\:_{j\in\:V}{x}_{ij}^{k}\:=\:\sum\:_{j\in\:V}{x}_{ji}^{k},\:\:\:\:\:\:\:\:\:\:\:\:\:\:\:\:\:\:\:\:\:\:\:\:\:\:\:\:\:\:\forall\:i\in\:V,\forall\:k\in\:K$$

The next set of constraints addresses capacity and load propagation. Equation (5) updates the vehicle load as it moves from node *i* to node *j*, accounting for the demand at the destination node. Equation (6) ensures that the load on a vehicle never exceeds its maximum capacity *Q* nor falls below zero, while Eq. (7) specifies that the initial load at the depot is zero for all vehicles.

5 $$\:{L}_{j}^{k}\ge\:{L}_{i}^{k}+\:{q}_{j}-Q.\:\left(1-\:{x}_{ij}^{k}\right),\:\:\:\:\:\:\:\:\:\:\:\:\forall\:\left(i,j\right)\in\:A\:\forall\:k\in\:K$$

6 $$\:0\le\:{L}_{i}^{k}\le\:Q,\:\:\:\:\:\:\:\:\:\:\:\:\:\:\:\:\:\:\:\:\:\:\:\:\:\:\:\:\:\:\:\:\:\:\:\:\:\:\:\:\:\:\:\:\forall\:i\in\:V,\forall\:k\in\:K\:$$

7 $$\:{L}_{0}^{k}=0,\:\:\:\:\:\:\:\:\:\:\:\:\:\:\:\:\:\:\:\:\:\:\:\:\:\:\:\:\:\:\:\:\:\:\:\:\:\:\:\:\:\:\:\:\:\:\:\:\:\:\:\:\:\forall\:k\in\:K$$

Time-window and service constraints are enforced through Eqs. (8) and (9). Equation (8) ensures that service at node *i* by vehicle *k* begins within the allowable time window [a.i., *bi*]. Equation (9) maintains temporal feasibility between consecutive visits, incorporating service time at the current customer and travel time to the next customer, with a big-M term used to deactivate the constraint when the arc is not traveled. Equation (10) sets the depot service start time as the time origin for each vehicle, anchoring all route times and preventing a free shift of $\:{T}_{i}^{k}$.

8 $$\:{a}_{i}\le\:{T}_{ij}^{k}\le\:{b}_{i},\:\:\forall\:i\in\:V,\forall\:k\in\:K$$

9 $$\:{T}_{j}^{k}\ge\:{T}_{i}^{k}+\:{s}_{i}+\:{t}_{ij}-{M}_{ij}\left(1-\:{x}_{ij}^{k}\right),\:\:\forall\:(i,j)\in\:A,\forall\:k\in\:K$$

10 $$\:{T}_{0}^{k}=0,\:\:\forall\:k\in\:K$$

With a tight choice $\:{M}_{ij}$= $\:{{b}_{i}+s}_{i}+{t}_{ij}-\:{a}_{j}$.

Fuel consumption and emissions are explicitly modeled in Eqs. (11) and (12). Equation (11) computes the fuel consumed by vehicle *k* on arc (*i*,*j*) as a function of the distance traveled and a load-dependent fuel rate that linearly interpolates between empty-load and full-load fuel consumption rates *ρ*0 and *ρ*1. Equation (12) calculates the CO_2_ emissions by multiplying the fuel consumed by the emission factor *γ*.

11 $$\:{F}_{ij}^{k}\ge\:{d}_{ij}\left[{\rho\:}_{0}+\left({\rho\:}_{1}-\:{\rho\:}_{0}\right)\frac{{L}_{i}^{k}}{Q}\right]-\:M\left(1-\:{x}_{ij}^{k}\right),\:\:\:\:\:\forall\:(i,j)\in\:A,\forall\:k\in\:K$$

12 $$\:{E}_{ij}^{k}=\:\gamma\:.\:{F}_{ij}^{k},\:\:\:\:\:\:\:\:\:\:\:\:\:\:\:\:\:\:\:\:\:\:\:\:\:\:\:\:\:\:\:\:\:\:\:\:\:\:\:\:\:\:\:\:\:\:\:\:\:\:\:\:\:\:\:\:\:\:\:\:\:\:\:\:\:\:\:\forall\:(i,j)\in\:A,\forall\:k\in\:K$$

To incorporate environmental policy constraints. Equation (13) sets a hard upper bound on the total emissions generated by the fleet. At the same time, Eq. (14) introduces aggregate distance *D* and aggregate emissions E (with non-negativity) to linearize the mean-emission-intensity constraint. Equation (15) enforces the fleet-wide cap on mean emission intensity (kg CO_2_ per km) using the aggregates from (14). Unlike conventional GVRP formulations that impose per-vehicle or absolute emission limits, the proposed constraints operate at the fleet level and include an emission-intensity policy that couples total emissions and total distance across all vehicles.

13 $$\:\sum\:_{k\in\:K}\sum\:_{\left(i,j\right)\in\:A}{E}_{ij}^{k}{x}_{ij}^{k}\:\le\:\:{E}_{max}$$

14 $$\:D=\:\sum\:_{k}\sum\:_{(i,j)}{d}_{i,j}{x}_{ij}^{k},\:E=\:\sum\:_{k}\sum\:_{(i,j)}{E}_{ij}^{k}{x}_{ij}^{k},\:D,\:E\ge\:0$$

15 $$\:E/D\le\:\eta_{max}$$

Finally, sub-tour elimination is achieved using the Miller–Tucker–Zemlin (MTZ) constraints given in Eqs. (16) and (17). Equation (16) prevents the formation of disconnected cycles that do not include the depot, and Eq. (17) defines the bounds and initialization for the auxiliary MTZ variables $\:{U}_{i}^{k}$. Equation (18) declares variable domains (binary routing decisions; non-negative continuous variables).

16 $$\:{U}_{i}^{k}-{U}_{j}^{k}+Q.\:{x}_{ij}^{k}\le\:Q-{q}_{j},\:\:\:\:\:\:\:\:\:\:\:\:\:\:\:\:\:\:\:\:\:\:\:\:\:\:\:\:\:\:\:\:\:\:\:\:\:\:\forall\:\left(i,j\right)\in\:A,\:i\ne\:0,j\ne\:0,\:\forall\:k\in\:K\:$$

17 $$\:{q}_{i}\le\:{U}_{i}^{k}\le\:Q,\:{U}_{0}^{k}=0,\:\:\:\:\:\:\:\:\:\:\:\:\:\:\:\:\:\:\:\:\:\:\:\:\:\:\:\:\:\:\:\:\:\:\:\:\:\:\:\:\:\:\:\:\:\:\:\:\:\forall\:i\in\:V,\forall\:k\in\:K$$

18 $$\:{x}_{ij}^{k}\in\:\left\{\mathrm{0,1}\right\},\:{T}_{i}^{k},\:{L}_{i}^{k},\:{F}_{ij}^{k},\:{E}_{ij}^{k},\:{U}_{i}^{k}\ge\:0$$

## Proposed solution approach

**Fig. 2:**
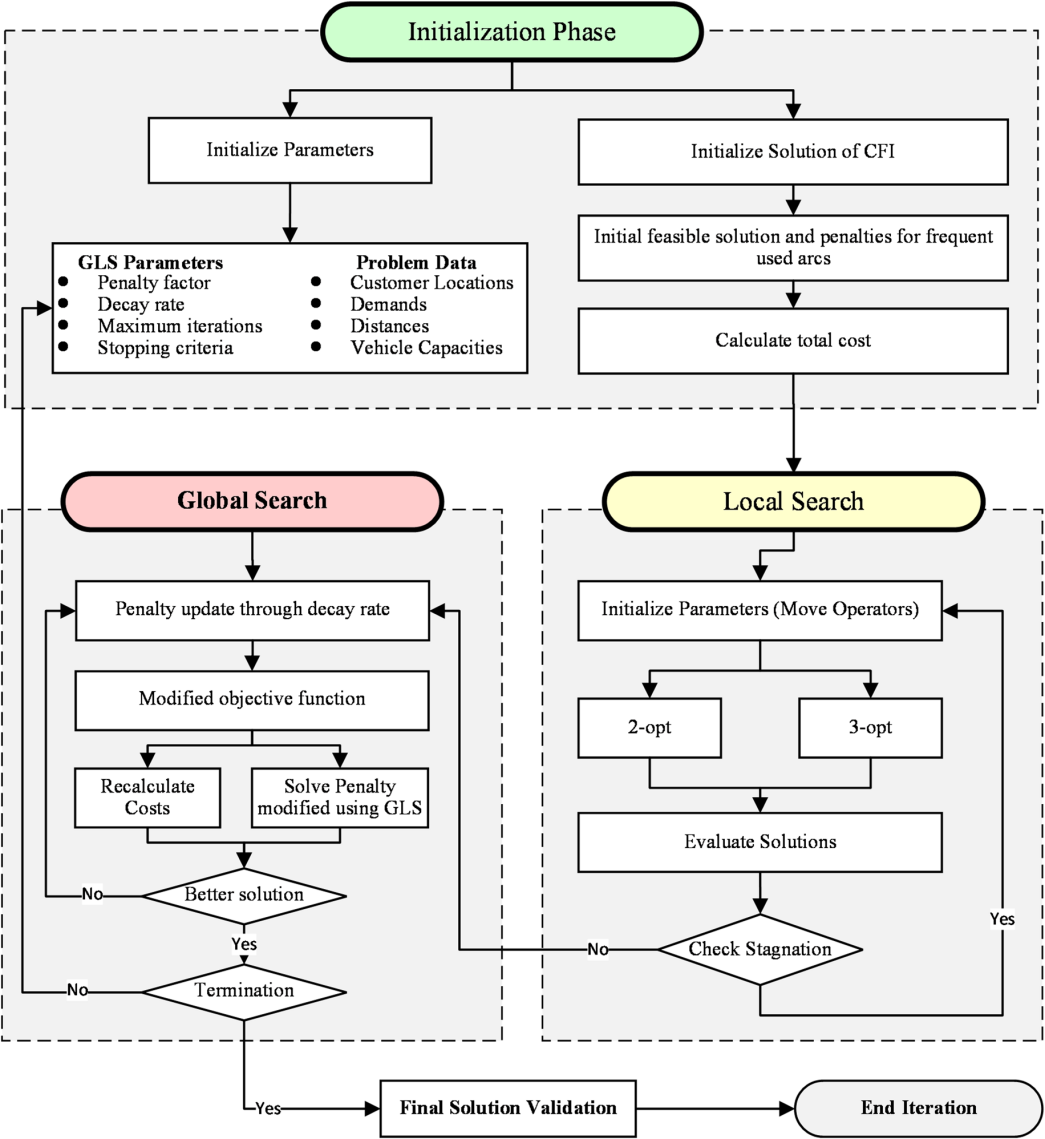
Three layered adaptive HGLS Flowchart.

The solution approach to solve the E-CVRPTW comprises three phases: (1) generating an initial seed solution via the CFI, (2) refining the solution using the GLS metaheuristic, and (3) integrating the two methods for iterative solution improvement (illustrated in Fig. 1). The proposed solution approach is termed Hybrid Guided Local Search (HGLS), and its pseudo-code is shown in Fig. 2.

### Cheapest feasible insertion (CFI) heuristic

We construct the initial solution with a CFI heuristic, a VRPTW adaptation of the classic cheapest-link idea. At each step, the method inserts the unserved customer and position that yields the smallest increase in the weighted fuel emissions objective while preserving feasibility (capacity, time windows, and per-vehicle distance limit). This procedure avoids premature cycles and naturally ensures that the in-degree and out-degree of every visited node are equal to 1.

i. **Cost evaluation**.

Let $\:\varPhi\:\left({L}_{i}^{k}\right)$ be the load-dependent fuel rate be defined in Sect. 3.2. The arc cost used during construction is; $\:{c}_{ij}^{k}=({w}_{1}+\:{w}_{2}\gamma\:)\varPhi\:\left({L}_{i}^{k}\right){d}_{ij}$, which is consistent with the model objective. For an insertion of customer *u* between consecutive nodes (*i*,* j*) in route *k*, we evaluate the exact marginal cost. By recomputing the route suffix from *i* onward (loads, arrival/start times, waiting, and distances). This accounts for the change in load and timing on all downstream arcs, not only (*i*,*u*) and (*u*,*j*).The marginal cost is defined as; $\:\varDelta\:C(u,r,\left(i,j\right),k)=\left(\sum\:_{affected\:arcs\:after\:insertion}{c}^{k}-\:\sum\:_{affected\:arcs\:before\:insertion}{c}^{k}\right)$.

ii. **Feasibility simulation**.

After a candidate insertion is identified, the evaluator verifies its feasibility by checking compliance with the model constraints defined in Sect. 3. This includes re-evaluating route load, service start times, time-window adherence, and fuel–emission totals using the same functional relationships and propagation rules already specified in the model. The insertion is considered feasible only if all relevant constraints, including the fleet-level emission-intensity policy, remain satisfied.

iii. **Capacity propagation (pickups)**.

For feasible insertions, the evaluator updates the route state by propagating load, arrival times, fuel consumption, and emissions along the remaining portion of the route. These updates follow directly from the load accumulation rule, the time-propagation constraint, and the fuel–emission functions defined in Sect. 3. This ensures that all downstream variables remain consistent with the modified route structure and ready for subsequent evaluations.

$\:{L}_{u}^{k}\leftarrow\:{L}_{i}^{k}+{q}_{u}$, add $\:{q}_{u}$ all downstream nodes *v* $\:{L}_{v}^{k}$ enforce $\:0\le\:{L}^{k}\le\:Q.$.

Time window with waiting:

$\:{A}_{u}^{k}\leftarrow\:{T}_{i}^{k}+{s}_{i}+{t}_{iu},\:{\:\:T}_{u}^{k}\leftarrow\:\mathrm{m}\mathrm{a}\mathrm{x}\{{a}_{u},\:{A}_{u}^{k}\}$, then propagate $\:{A}_{j}^{k}\leftarrow\:\:{T}_{u}^{k}+{s}_{u}+{t}_{uj},\:{\:\:T}_{j}^{k}\leftarrow\:\mathrm{m}\mathrm{a}\mathrm{x}\{{a}_{j},\:{A}_{j}^{k}\}$, and continue to the route end, checking $\:{T}_{v}^{k}\le\:{b}_{v}$ for all downstream *v*. A candidate is admissible if these checks pass.

### Route opening and selection policy

CFI maintains a set of partial routes. While unserved customers remain:

1. For every unserved customer *u*, for every current route *k*, and for every insertion position (*i*,*j*) in that route, compute feasibility and $\:\varDelta\:{\Phi\:}$.
2. Select the admissible insertion with minimum $\:\varDelta\:{\Phi\:}$. Tie-break by (i) smaller time-window slack at *u*, $\:{b}_{u}-\:{T}_{u}^{k},$ (ii) smaller added distance (iii) smaller route load for insertion.
3. If no admissible insertion exists in any open route and unused vehicles remain, open a new route 0 $\:\to\:$ *u*$\:\:\to\:$ 0 at the best position (evaluated identically).
4. If no unused vehicle remains, the construction process terminates and the partial solution is passed to the improvement phase. Under normal operation, the CFI operator continues attempting customer insertions until all customers are assigned to routes, provided that the available number of vehicles *m* is sufficient to serve the total demand while satisfying capacity, time-window, and environmental constraints.

**Fig. 3:**
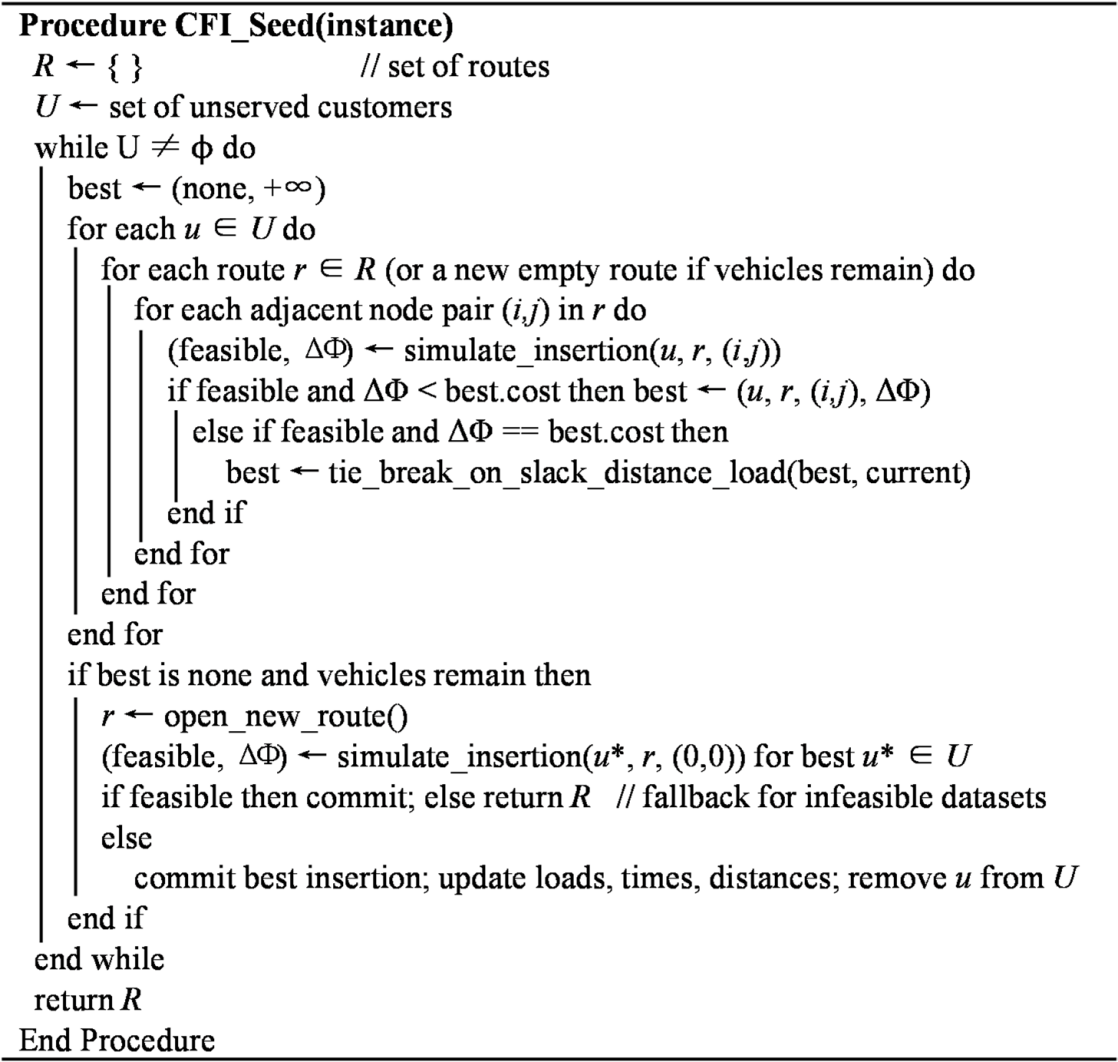
Pseudocode of CFI.

Because CFI inserts into existing depot-anchored routes, in/out degree of visited nodes remains one, and no sub-tours can form during construction. If a customer cannot be feasibly inserted into any open route at a given iteration, the CFI procedure does not discard the customer. Instead, such unassigned customers are deferred to a secondary reconstruction step, where additional insertion attempts are made using any remaining vehicle capacity or, if applicable, by opening a new route. This mechanism guarantees that all customers are reconsidered exhaustively before the construction phase passes its partial solution to the improvement phase. The pseudo-code for CFI is presented in Fig. 3.

### Guided local search

**Fig. 4:**
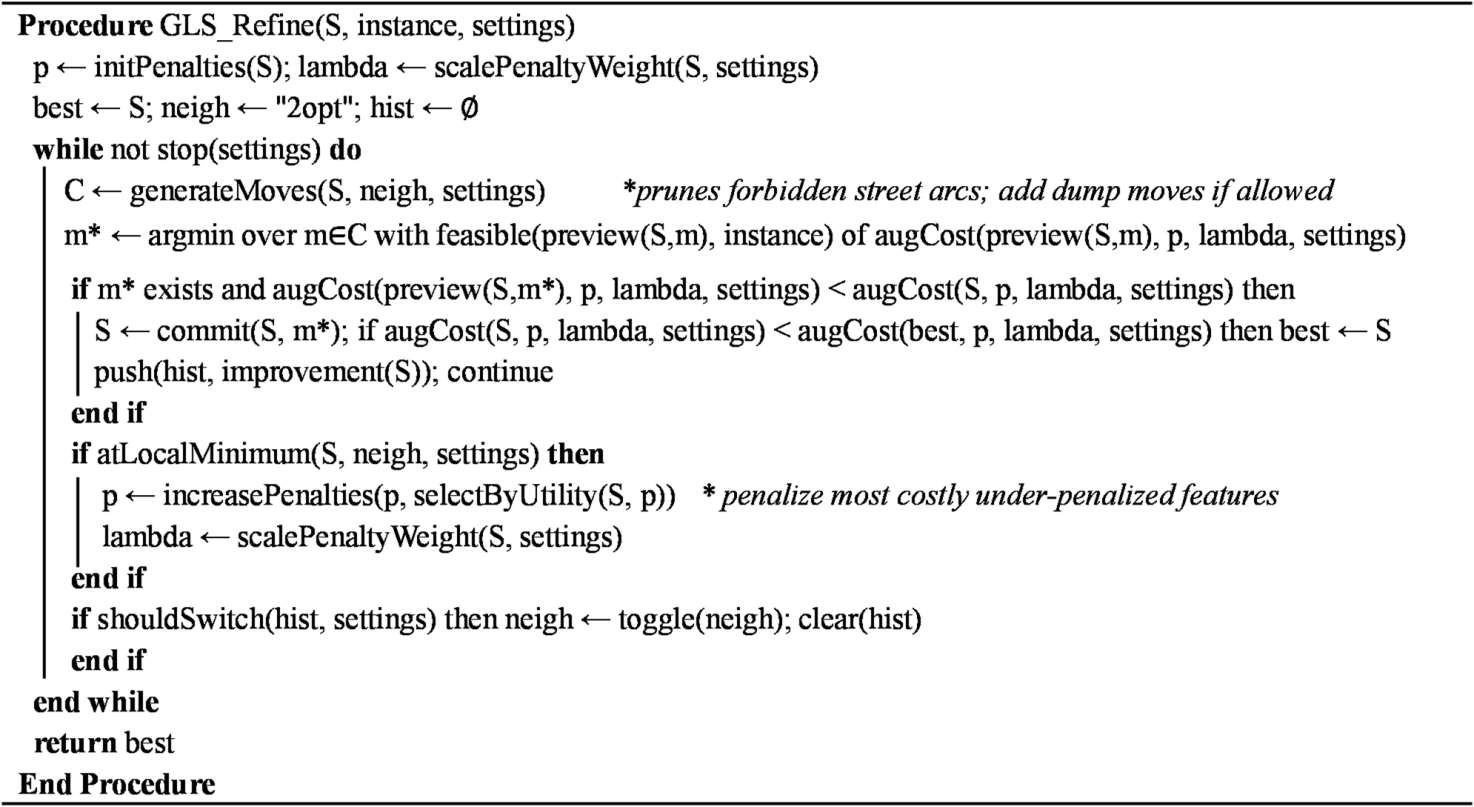
Pseudocode of the proposed HGLS algorithm.

The seed solution is refined with GLS, which augments local search with feature penalties to escape local minimum while preserving feasibility (vehicle capacity, time windows, and per-vehicle distance limits). The pseudocode of the proposed HGLS algorithm is presented in Fig. 4.

#### Exploration (neighborhoods, feasibility, acceptance)

In GLS, solution improvement occurs by exploring a neighborhood, which is a set of candidate solutions generated by modifying the current route structure. Each modification, called a move operator, changes the customer order or route composition and is then evaluated against feasibility rules (vehicle capacity, time windows, and maximum route length). GLS systematically tests these moves and accepts the one that yields the most significant reduction in the penalty-augmented objective, ensuring that every accepted move both improves solution quality and remains feasible. The neighborhood structure in our implementation includes the following standard VRP move operators:

- 2-opt (intra-route): reverse a segment within a route to remove crossings and reduce travel.
- 3-opt (intra-route): remove three edges and reconnect to enable deeper restructuring.
- Relocate (inter-route): move one customer to a different position/route.
- Swap (inter-route): exchange two customers across routes.

After each candidate moves, we recompute affected suffixes (loads, arrival/start times with waiting, and cumulative distance) and enforce feasibility. A move is accepted if it strictly improves the augmented objective and remains feasible. We use best-improving selection by default; first-improving is permitted on the most significant instances to reduce runtime. The search starts with 2-opt for fast geometric cleanup. If the best relative improvement over a short sliding window falls below 1%, we switch to the “3-opt + inter-route” neighborhood set (relocate/swap). When improvement resumes, we return to 2-opt.

#### Features and the penalty-augmented objective

Let ℱ (S) be the set of features in solution S. We take directed arcs (*i*,*j*) as features by default. Base cost of arc feature (*i*,*j*):$\:{r}_{ij}=\:\sum\:_{k\in\:K}{c}_{ij}^{k}{x}_{ij}^{k}$, (see Sect. 3.2 for the $\:{c}_{ij}^{k}\:$explanation). GLS minimizes the penalty-augmented objective.

$${{\Phi }}\left( S \right) = \underbrace {\mathop \sum \limits_{\left( {i,j} \right) \in A} {r_{ij}}}_{fuel - emission\;objective} + \;\lambda \left( {\underbrace {\mathop \sum \limits_{\left( {i,j} \right) \in {\mathcal{F}}\left( {\mathrm{S}} \right)} {p_{ij}}}_{arc\;penalties} + \underbrace {\mathop \sum \limits_{\left( i \right) \in V\left\{ 0 \right\}} {p_i}}_{optional\;node\;penalties}\;} \right)$$

where *p*_*ij*_ ≥ 0 and $\:\lambda\:>0$ is the penalty weight.

#### Penalty weight scaling and update rule

We set the penalty weight dynamically:

$$\:\lambda\:=\:\alpha\:\frac{{{\Phi\:}}_{0}\left({S}^{*}\right)}{\mathcal{F}\left({S}^{*}\right)}$$

where $\:{{\Phi\:}}_{0}\left({S}^{\mathrm{*}}\right)$ is the unpenalized objective at a local minimum $\:{S}^{\mathrm{*}}$, $\:\left|\mathcal{F}\left({S}^{\mathrm{*}}\right)\right|$ is its feature count, and *α* ∈ [0.2,0.5] is instance independent. At each local minimum, compute utilities for features present in $\:{S}^{\mathrm{*}}$: $\:{u}_{ij}=\:\frac{{r}_{ij}}{1+\:{p}_{ij}}$. Identify all features attaining the maximum utility and increase their penalties by one: $\:{p}_{ij\leftarrow\:}{p}_{ij}+1$. This drives the search away from expensive, under-penalized substructures while remaining consistent with the fuel emissions objective.

#### Consistency with the model and case study

GLS evaluates every candidate move under the same feasibility rules as the mathematical model and accepts a move only if all referenced constraints remain satisfied routing/flow (Constraints (2)–(4)), load and capacity propagation (Constraints (5)-(6)), time windows with waiting and temporal precedence (Constraints (7)–(9) with tightened *Mij*, and the per-vehicle distance limit (Constraint (16); depot closing if used). For the municipal waste case study, any street/approach rules (e.g., side-of-street, turn bans) defined in the case-study data are enforced by excluding prohibited arcs from neighborhood generation; candidates with (*i*, *j*) in the forbidden set are never proposed. If transfer-station visits are modeled as regular nodes in the constraints, GLS treats them like any other node; if they are not modeled, GLS does not introduce them.

To ensure reproducibility, Table 1 reports the main parameter settings used for the proposed HGLS in all experiments. Unless stated otherwise, the same configuration is applied across all instances and datasets. These parameters control the penalty-based diversification mechanism of Guided Local Search, the local search neighborhoods, and the termination rule.

**Table 1:** Main parameter settings of the proposed HGLS.

Parameter	Setting
Feature definition	Arc based features (i, j)
Penalty weight	λ computed using standard GLS scaling with fixed α = 0.30
Penalty update	At each local minimum, increment *p*_*ij*_ by 1 for features attaining the maximum *u*_*ij*_
Local search operators	Relocate, Swap, 2 opt
Stopping rule	A fixed time limit per run (kept constant within each benchmark set), with early stopping after 500 non-improving iterations

## Experimental analysis

In the absence of established benchmarks tailored to emission-aware routing, we assess the proposed method on the community’s standard VRPTW test problems. This allows us to isolate the routing capability of our approach under widely accepted conditions, before activating environmental terms in the case study. All benchmark runs follow the canonical lexicographic criterion: (i) minimize the number of vehicles (NV); (ii) among solutions with identical NV, minimize total routed distance. Environmental terms (fuel and CO_2_) are not used on these benchmarks. We employ the Solomon instances (families C, R, RC, 100 customers) and the Gehring & Homberger (GH) extensions (200–1000 customers). Coordinates are interpreted in kilometers; when travel times are not provided, unit speed is assumed (time = distance). Early arrivals wait at service start without penalty. Depot opening time is 0; any depot closing time present in the file is enforced. All methods: proposed by HGLS, GA, and SA do use the same evaluator (capacity along the route suffix, time windows with waiting, and depot return, if specified). Following common VRPTW benchmarking practice, the HGLS was run with a size dependent CPU time budget of 1800 s for instances with *n* ≤ 201, and 3600 s for 201 < *n*≤801, with early termination activated if no improvement is observed for 500 consecutive non improving iterations.

### Benchmark evaluation on Solomon

HGLS is run in NV-first mode: any feasible move that reduces NV is accepted immediately; distance is then optimized at the fixed NV using 2-opt/3-opt (intra-route) and relocate/swap (inter-route) with Guided Local Search penalties. Results are reported in Table 1. For each instance, we list the best-known NV and distance (BKS), our NV and distance, a distance gap computed only when NV matches, and the GA/SA distances for reference. To make lexicographic dominance transparent, we annotate our NV with * when fewer than BKS (primary improvement) and with † when more than BKS (primary degradation). When NV differs, the distance gap is not comparable and is shown as “–”. The BKS values are taken from the SINTEF TOP VRPTW repository and cross checked against the published Solomon results reported in Electronics^47^; SINTEF reports BKS under a hierarchical objective that minimizes the number of vehicles first and then total distance, using double precision Euclidean distances rounded to two decimals.

**Table 2:** HGLS performance against BKS, GA, and SA.

Problem	BKS NV	BKS Dist.	NV (HGLS)	Dist. (HGLS)	Time (s)	Gap% @ same NV	GA Dist.	Time (s)	SA Dist.	Time (s)
C101	10	828.94	10	828.94	32	0.00	830.20	41	830.20	47
C102	10	828.94	10	828.94	33	0.00	860.50	43	860.50	40
C103	10	828.06	10	828.06	34	0.00	862.80	39	862.80	43
C104	10	824.78	10	864.22	32	4.56	880.20	41	880.20	65
C105	10	828.94	10	828.94	32	0.00	840.50	45	840.50	52
C106	10	828.94	10	828.94	33	0.00	845.20	45	845.20	54
C107	10	828.94	10	828.94	33	0.00	842.20	45	842.20	59
C108	10	828.94	10	854.31	32	3.06	861.10	50	861.10	73
C109	10	828.94	10	854.78	35	3.11	870.20	50	870.20	75
C201	3	591.56	3	591.56	29	0.00	615.30	36	615.30	65
C202	3	591.56	3	591.56	28	0.00	620.40	34	620.40	53
C203	3	591.17	3	591.17	37	0.00	617.40	39	617.40	58
C204	3	590.60	3	590.60	29	0.00	634.40	42	634.40	67
C205	3	588.85	3	588.85	37	0.00	605.40	44	605.40	64
C206	3	588.49	3	588.49	25	0.00	623.40	48	623.40	61
C207	3	588.29	3	588.29	36	0.00	629.33	51	629.33	65
C208	3	588.32	3	588.32	38	0.00	614.90	48	614.90	64
R101	20	1637.70	19*	1656.60	55	–	1709.0	75	1709.50	80
R102	18	1466.60	18	1466.60	48	0.00	1535.30	72	1535.30	85
R103	14	1208.70	14	1208.10	45	0.00	1305.20	70	1305.20	82
R104	11	976.61	10*	1010.60	57	–	1055.50	73	1055.50	88
R105	15	1355.30	14*	1395.70	58	–	1445.60	75	1445.60	70
R106	13	1234.60	13	1234.60	48	0.00	1298.20	78	1298.20	75
R107	11	1064.60	11	1103.80	50	3.68	1145.80	75	1145.80	90
R108	10	938.20	10	966.99	59	3.07	999.20	75	999.20	80
R109	13	1146.90	12*	1200.20	52	–	1295.80	78	1295.80	75
R110	12	1068.00	11*	1127.30	52	–	1200.50	64	1200.50	85
R111	12	1048.70	11*	1096.40	52	–	1165.20	58	1165.20	96
R112	10	953.63	10	953.63	45	0.00	998.70	70	998.70	68
R202	8	1034.40	8	1034.40	40	0.00	1110.50	53	1107.20	60
R203	6	874.87	6	874.87	39	0.00	910.50	44	925.50	80
R204	5	735.80	4*	735.80	38	–	855.50	30	894.20	75
R205	5	954.16	5	1036.50	49	8.63	1125.20	65	1110.20	95
R206	4	879.86	4	879.86	38	0.00	1175.60	33	1130.80	70
R207	4	797.99	4	876.22	57	9.80	1118.40	65	1162.70	95
R208	4	705.33	4	760.22	36	7.78	930.70	33	967.60	90
R209	5	859.39	4*	859.39	53	–	994.20	61	1037.20	95
R210	6	905.21	2*	942.94	45	–	1090.20	70	1110.80	89
R211	4	753.15	4	790.21	40	4.92	1041.20	62	996.30	95
RC101	14	1619.80	16†	1656.60	45	–	1740.10	63	1805.55	98
RC102	14	1457.40	13*	1532.30	46	–	1665.20	47	1617.20	93
RC103	11	1258.00	12†	1344.00	55	–	1458.10	68	1509.20	91
RC104	10	1135.50	10	1135.50	40	0.00	1435.20	52	1518.80	95
RC105	15	1513.70	14*	1662.00	45	–	1750.20	70	1775.50	88
RC106	13	1378.00	12*	1344.00	42	–	1555.20	68	1610.20	96
RC107	12	1212.80	12	1263.10	53	4.15	1410.70	65	1418.40	95
RC108	11	1117.50	11	1144.90	40	2.46	1375.08	39	1438.20	83
RC201	9	1261.80	9	1261.80	44	0.00	1438.02	42	1515.20	72
RC202	8	1095.60	8	1095.60	51	0.00	1308.70	55	1371.40	85
RC203	5	926.82	5	926.82	32	0.00	1350.20	50	1318.70	80
RC204	4	786.38	10†	854.31	40	–	1005.20	60	1054.20	90
RC205	7	1157.60	7	1180.70	38	1.99	1317.60	58	1371.20	88
RC206	7	1054.60	4*	1114.80	40	–	1254.20	60	1334.40	90
RC207	6	966.08	6	966.08	35	0.00	1218.20	45	1238.20	85
RC208	4	778.93	4	778.93	43	0.00	932.20	52	973.70	71

Three consistent patterns emerge. (i) On clustered (C) instances, HGLS matches the best-known NV throughout and typically reproduces the best-known distance; when not exact (e.g., C104, C108, C109), deviations are modest, as expected when opportunities to reduce vehicles are limited. (ii) On random (R) instances, HGLS frequently collapses routes, e.g., R101, R104–R105, R109–R111, R204, and R209–R210, yielding lexicographic improvements even when distance rises slightly. When NV matches (R102, R106, R112), distances remain at or extremely close to the references. (iii) On mixed (RC) instances, HGLS attains several NV reductions (e.g., RC106, RC206), many NV matches with small gaps, and a small number of degradations (RC101, RC103, RC204) that are consistent with runs where NV-first acceptance or per-vehicle distance limits were not fully aligned with the benchmark standard.

Across the 55 Solomon instances, HGLS reduces NV on 13, matches NV on 39, and is higher on 3. When NV matches (39 cases), the distance gap has a median of 0.00%, a mean of 1.47%, a range of [0.0-%, 9.80%], and an interquartile range of [0.00%, 2.76%]. By family, the median gap is 0.00% throughout; mean gaps are 0.64% (C), 2.91% (R), and 0.95% (RC). Against GA and SA, HGLS attains a lower distance in 55/55 instances (same evaluator), indicating consistently more vigorous route polishing at fixed NV. The three RC degradations are outliers; they typically disappear when enforcing explicit NV-first acceptance and avoiding overly restrictive per-vehicle distance caps (see the robustness section of the methodology).

The reported computational times reflect the relative complexity of the three solution approaches. Across all instance classes, the proposed HGLS consistently exhibits the lowest runtime, owing to its single-solution search strategy combined with guided penalties and an efficient CFI-based construction phase. GA requires additional time due to population-based evaluations, while SA is generally slower because of its cooling schedule and repeated acceptance checks. Variations across instances are mainly influenced by problem structure, route fragmentation, and early termination of infeasible insertions, particularly in R-type instances.

### Large-scale evaluation on GH

To probe scalability, we also evaluate selected Gehring & Homberger instances with 200, 400, and 800 customers (ten per size). Here, we compare HGLS with SA using the same evaluator. Table 2 reports distances, NV, and the percentage improvement of HGLS vs. SA.

**Table 3:** HGLS performance against gehring and Homberger 200, 400, and 800 customer instances.

Nodes	Instance	HGLS Dist.	NV	SA Dist.	NV	Gap% vs. SA
200	R1_2_1	4803.41	20	4903.41	20	−2.08
200	R1_2_2	4149.27	18	4249.27	19	−2.41
200	R1_2_3	3452.92	18	3852.92	19	−11.58
200	R1_2_4	3146.42	18	3446.42	18	−9.54
200	R1_2_5	4124.10	18	4484.10	19	−8.73
200	R1_2_6	3602.42	18	3972.42	19	−10.27
200	R1_2_7	3186.50	18	3536.50	19	−10.98
200	R1_2_8	2971.92	18	3471.92	18	−16.82
200	R1_2_9	3782.52	18	4062.52	19	−7.40
200	R1_2_10	3317.65	18	3917.65	18	−18.09
400	R_1_4_1	10392.96	40	10942.96	41	−5.29
400	R_1_4_2	8969.84	38	9869.84	38	−10.03
400	R_1_4_3	7892.26	37	8992.26	37	−13.94
400	R_1_4_4	7312.02	36	8282.02	36	−13.27
400	R_1_4_5	9292.52	36	10222.52	37	−10.01
400	R_1_4_6	8389.88	36	9289.88	37	−10.73
400	R_1_4_7	7660.53	36	9089.53	36	−18.65
400	R_1_4_8	7290.53	36	8690.53	36	−19.20
400	R_1_4_9	8703.00	36	9763.00	37	−12.18
400	R_1_4_10	8116.08	36	9286.08	37	−14.42
800	R1_8_1	37005.00	81	39005.00	81	−5.41
800	R1_8_2	33410.00	73	37410.00	74	−11.97
800	R1_8_3	30549.82	72	34549.82	73	−13.09
800	R1_8_4	28200.00	72	32200.00	72	−14.18
800	R1_8_5	34130.00	72	39130.00	73	−14.65
800	R1_8_6	31394.00	72	36394.00	72	−15.93
800	R1_8_7	29156.00	72	34556.00	72	−18.52
800	R1_8_8	27932.00	72	31332.00	72	−12.17
800	R1_8_9	32621.00	73	37721.00	73	−15.63
800	R1_8_10	31100.00	72	37300.00	72	−19.94

Across sizes, HGLS delivers lower distances than SA in every case, often by double-digit percentages, while maintaining the same or fewer vehicles. This confirms that the search remains stable and effective as the instance size grows from 200 to 800 customers. HGLS improves distance relative to SA on 30/30 GH instances, with mean percentage improvements of 9.79% (200), 12.77% (400), and 14.15% (800); medians are 9.90%, 12.72%, and 14.42%, respectively. Vehicle usage is never worse than SA: HGLS uses fewer NV on 6/10 (200), 5/10 (400), 3/10 (800), and equal NV on the remainder. Aggregated over all 30 instances, the mean improvement vs. SA is 12.24%, with 14 cases using fewer vehicles and 16 equal.

On Solomon, HGLS matches the best-known vehicles on clustered instances and frequently reduces the number of vehicles on random/mixed instances, while remaining close to the best-known distance whenever NV matches. On GH, the method scales to 800-customer problems with consistent advantages over SA. Together, these results demonstrate that the proposed approach is competitive under the community’s distance–vehicles standard and is suitable for large-scale applications. The subsequent case study reactivates environmental terms to assess fuel, CO_2_, and emission intensity under municipal-waste operating conditions.

## Case study

Water and sanitation services Peshawar (WSSP) operate solid-waste collection across four service zones (A–D) for a metropolitan population of approximately 4.3 million. This study focuses on Zone D, a dense urban district with 109 containerized pickup points distributed across a heterogeneous street network that includes one-way streets, narrow alleys, signed turn restrictions, and school-zone time restrictions. As shown in Table 3, the legacy operation deploys 36 Suzuki compactors (nominal capacity 6 m^3^) whose daily routes are sequenced ad hoc despite the availability of GPS traces. Field audits show repeated traversals of a small set of arterials, avoidable deadheading between noncontiguous clusters, and a persistent mismatch between vehicle capacity and container volumes on access-constrained streets. The cumulative effect is elevated distance, fuel use, and CO_2_ emissions, alongside a noticeable concentration of near-deadline arrivals at several commercial sites with tight opening hours.

The collection system is pick-up-oriented: vehicle load increases after each serviced container. Containers are of two types and volumes: 2.0 m^3^ arm-roll and 0.8 m^3^ small and are fixed to their coordinates. Each site has an operational time window (typical span 08:00–16:00) and a measured service time that includes approach, positioning, hooking/emptying, and reseating; empirical values range from 46 to 60 min for arm-roll units and 40–56 min for small containers. Early arrivals are permitted to wait without penalty until the window opens. Vehicles start at a single depot at 08:00 and must return by 16:30; when enforced by duty policy, a per-route distance limit of 35 km caps daily exposure.

The street network is constructed from the operator’s GIS export and projected to a local planar system for accurate distance measurement. Access rules are embedded in the graph: compactors are prohibited from alleys and weight-restricted links; specific approaches enforce side-of-street service; and arcs adjacent to schools are closed during 07:30–08:30 and 13:00–14:00. These prohibitions remove the corresponding arcs from the feasible movement set so that the algorithm never proposes infeasible maneuvers. Travel times are derived from corridor-level GPS averages when available; otherwise, times are taken proportional to distance, with explicit waiting allowed. Fuel and emissions are central to the case. The fuel rate varies with carried load according to a simple linear load-dependent law calibrated for this fleet, with ρ₀ = 1.00 L/km at empty load and ρ₁ = 2.00 L/km at full load. Carbon emissions are computed from fuel via the factor γ = 2.32 kg CO_2_/L. Costs are reported at $1.00/L for fuel and $0.08/kg for CO_2_ emissions. In addition to minimizing fuel and CO_2_ jointly, the city has articulated two policy targets that must be respected in planning: a carbon budget set at 85% of the reconstructed status-quo emissions for the same service day, and an emission-intensity ceiling of 2.0 kg CO_2_ per kilometer. These targets shape the feasible solution space and define compliance in the subsequent analysis.

Two operational configurations are examined to reflect current assets and the planned transition. The current fleet configuration treats all vehicles as 6 m^3^ compactors (36 units). The small-vehicle configuration represents the proposed deployment on access-constrained streets with 3 m^3^ vehicles (50 units). Treating each configuration as homogeneous preserves a consistent capacity representation across routes while allowing a clean comparison of routing structure, distance, fuel, and emissions under different equipment strategies. Within this setting, the planning problem is to assign vehicles to pick up points and sequence the visits so that every site is serviced exactly once within its time window; vehicles depart from and return to the depot; cumulative pickups never exceed vehicle capacity at any point in a route; street-level access rules are never violated; and the resulting routes jointly minimize a weighted measure of fuel and CO_2_ while satisfying the carbon-budget and intensity targets. The evaluation reports operational outcomes (number of vehicles used, total distance, waiting statistics), environmental outcomes (total fuel, total CO_2_, and CO_2_ per kilometer), and economic outcomes (fuel, carbon, and combined costs).

### Data collection and preprocessing

We assembled the Zone D instance from four operational streams: a GIS export of the street network and pickup locations, fleet GPS traces, the vehicle registry (capacity and access tags), and service logs (per-stop times and windows). All times are expressed in minutes from the daily start; distances are in kilometers. The GIS graph was projected to a local planar system and enriched with directionality and turn restrictions. GPS trajectories were map-matched, outliers removed (median-absolute-deviation filter), and link speeds estimated by corridor/time-of-day. Where telemetry was sparse, travel time was set proportional to length; early arrivals wait. Operating rules (one-ways, turn bans, alley width/weight limits, and side-of-street approaches) were encoded by removing forbidden arcs. This yields vehicle-specific directed graphs in which compactors and small vehicles inherit different feasible movements. Time-dependent closures (e.g., school-zone windows) were applied as arc unavailability intervals to ensure that infeasible candidates are never generated.

All 109 pickup points were validated and the vehicles plus bins data is given in Table 3. Site windows from operations were harmonized and converted to the unified clock; service times were taken from logs, with missing entries imputed by container-type medians and truncated to plausible bounds. Demands reflect daily pickup volumes per container type; the instance follows the pickup convention (vehicle load increases after service). To match the single-capacity representation used in the optimization, we emitted two homogeneous files: current-fleet and small-vehicle, consistent with the configurations (see Sect. 5.1). Duty distance limits and depot hours are embedded at the instance level and enforced by the evaluator. The load-dependent fuel law and emission factor are embedded in the arc cost evaluator; no additional tunable are introduced at this stage. Policy targets (carbon budget and intensity ceiling) are stored alongside instance metadata for automatic compliance checks during solution. Before optimization, we verified: (i) reachability of every site by at least one compatible vehicle within its window; (ii) consistency of distances/times (shortest-path vs. direct edges); (iii) capacity feasibility under the pickup convention along route suffixes; and (iv) stable time propagation by using tight precedence margins derived from site latest/earliest times and measured service durations (waiting allowed) (Table 4).

**Table 4:** Vehicles and bins data.

Category	Type	Capacity	Details
Vehicles	Suzuki Compactors	6 m^3^	Used for collecting waste from bins across Zone D.
	Suzuki vehicles	3 m^3^	Smaller capacity vehicles assigned based on bin compatibility.
Bins	Arm roll containers	2 m^3^	Larger bins require vehicles with suitable capacities for efficient collection.
	Small containers	0.8 m^3^	Smaller bins are placed in specific locations for waste collection by compatible vehicles.

The data in Table A1 in appendix provides a detailed overview of vehicle routing and scheduling problem, highlighting key parameters such as geographical coordinates, service time windows, demand volumes, and service durations for each point in the network. It illustrates a wide range of demands (cubic meters) with heterogeneous vehicles, having service windows spanning from early morning (8:00 AM – 4:00 PM).

### Experimental design and evaluation

#### Policy scenarios

We structure the case study around six scenarios that together expose the operational and environmental behavior of Zone D under realistic policy levers. All scenarios share the same instance (data, access rules, time windows, pickup loads) and are solved with the same evaluator and stopping criteria; only the policy posture, objective weighting, and regulatory constraints change. This design ensures that differences in outcomes can be attributed to policy choices rather than data or algorithmic artifacts. Each scenario is run under both fleet configurations (current-fleet 6 m^3^; small-vehicle 3 m^3^).

i. **S**_**0**_: **Status-quo replication**.

S_0_ reconstructs the operator’s current day to establish a reference point for all subsequent comparisons. We replay observed sequences and break ties using shortest-path completion consistent with access rules and time windows. No optimization is performed; the output is the baseline set of routes and their metrics: number of vehicles used (NV), total distance *D*, total fuel ∑*F*, total CO_2_ ∑*E*, and emission intensity *E* /*D*. These quantities also provide the normalization factors used later when interpreting trade-offs. S0 is not intended to be competitive; it serves as an anchor that reflects current practice.

ii. **S1: Fuel-only optimization**.

S_1_ is the operational efficiency posture. The scalar objective emphasizes fuel exclusively, which, through the load-dependent fuel law, implicitly discourages long arcs under high load, excessive deadheading, and unnecessary re-entries into dense clusters. Because emissions are proportional to fuel, S1 is expected to reduce ∑*E* as a by-product; the extent of reduction depends on how much high-load distance the optimizer can eliminate without violating access and time-window constraints. S1 isolates the benefits achievable solely through routing when management’s primary lever is fuel expense.

iii. **S2: Balanced trade-off**.

S_2_ sets symmetric weights for fuel and emissions to reflect equal valuation of costs and carbon. This produces routes that reduce the distance traveled during heavy loads while also smoothing load profiles and timing to avoid bursts of high-emission travel. Compared with S_1_, we expect slightly longer *D* when small increases in distance buy a disproportionate reduction in ∑*E* (e.g., by shifting load earlier to lighter segments), and slightly lower *E*/*D* (cleaner kilometers). S2 represents a pragmatic middle ground for agencies that must report both financial and environmental performance.

iv. **S3: Emissions-focused optimization**.

S_3_ places full emphasis on emissions. In this posture, the search prioritizes reductions in ∑*E* even when the implied fuel savings are modest. Because the emission factor converts liters to kg CO_2_, pure emissions emphasis will still drive many of the same structural improvements as S1 (shorter heavy-load arcs), but S_3_ tends to prefer solutions that reduce emission intensity on the margin, e.g., by re-sequencing to serve heavier customers when they are closer, at the cost of slightly longer empty re-positioning. S_3_ is informative for agencies preparing climate inventories or testing aggressive de-carbonization stances.

v. **S4: Carbon-budget compliance**.

S_4_ embeds a collection-day fleet-level carbon budget: total collection-day emissions must not exceed a fixed budget set as a fraction of the status-quo day. Within this feasible set, the objective weights are chosen by the knee-point procedure (Sect. 5.3.2), so the reported solution is not merely “any feasible plan,” but the one that lies on a near-Pareto part of the frontier subject to the budget. We anticipate (i) a modest increase in distance relative to S1 when the budget binds, (ii) a meaningful drop in *E*/*D*, and (iii) substitution effects between fleet configurations (the small-vehicle configuration should satisfy the budget with less operational sacrifice).

vi. **S5: Emission-intensity compliance**.

S_5_ targets the quality of kilometers: the ratio *E*/*D* must not exceed a stated ceiling. This cap is operationally attractive because it is robust to day-to-day fluctuations in total workload and makes route “cleanliness” auditable. As in S_4_, the knee-point procedure sets weights, but is subject to the intensity cap. Solutions typically smooth load across routes and pull heavy customers inward (served when closer), which reduces variance in route intensities and benefits congested corridors. Where S_4_ ensures an absolute carbon reduction, S5 ensures that each kilometer remains within a sustainable emission envelope.

#### Weight selection and sensitivity for w1, w2

This subsection specifies how we choose the fuel emissions weights and why the choice is defensible. The procedure is data-driven, reproducible, and stress-tested for robustness; it is applied identically to both fleet configurations. We explore a grid of weights, trace the empirical trade-off between fuel and CO_2_, pick the knee point (the most efficient compromise), check it against an economic anchor, and then verify stability by sensitivity analysis on the fuel and emissions parameters.

##### Step 1

*Normalization and grid exploration*.

To avoid scale bias, we normalize each objective by the status-quo (S_0_) values:

$$\:\widehat{F}\left({w}_{1}\right)=\frac{\sum\:F\left({w}_{1}\right)}{\sum\:{FS}_{0}}\:,\:\widehat{E}\left({w}_{2}\right)=\:\frac{\sum\:E\left({w}_{2}\right)}{\sum\:{ES}_{0}}\:$$

With *w*_1_ = 1 − *w*_2_. We solve the instance for *w*_2_ ∈ (0, 0.05… 1). Adjacent runs are warm-started from the previous solution to ensure the observed trade-off is not a by-product of different convergence histories.

Outputs per weight. We record $\:\widehat{F}$, $\:\widehat{E}$ distance *D*, vehicles used (NV), intensity *E*/*D*, and total cost (fuel + carbon). Multiple independent seeds (≥ 5) are run per weight; we retain the best objective value and report dispersion across seeds.

##### Step 2

*Knee-point selection (primary choice)*.

We interpret the set $\:\left\{\widehat{F}{(w}_{1}\right)$, $\:\widehat{E}{(w}_{2})$} as a discrete Pareto-like curve and select the knee by maximum curvature. Discrete curvature is computed using centered triples ($\:{w}_{2}^{-}$, $\:{w}_{2},\:{w}_{2}^{+}\:$); if several weights tie, we prefer the one with lower intensity *ED* and then lower distance *D*. The knee is our default (*w*1, *w*2) for policy-neutral reporting and a starting point for policy scenarios.

##### Step 3

*Economic cross-check (price anchor)*.

Because emissions are proportional to fuel, the per-liter effective cost is: p_f_​ + γ_pc​_ = 1.00 + 2.32 × 0.08 = 1.186 USD/L. We verify that the knee’s routes are consistent with this valuation. If the knee and the price-anchored weight led to materially different solutions (e.g., ≥ 2% deviation in both $\:\widehat{F}$ and $\:\widehat{E}$), we report both and discuss their managerial interpretation (cost-centric vs. compromise-centric). In subsequent sections, the main figures highlight the knee; the appendix includes the price-anchored results.

##### Step 4

*Policy-consistent refinement (for S4*,* S5)*.

When a carbon budget or intensity ceiling binds, we choose the smallest $\:{w}_{1}\widehat{F}+\:{w}_{2}\widehat{E}$on the feasible boundary (i.e., the shallowest supporting slope that satisfies the cap). Practically, we sweep the grid, keep solutions with *E* ≤ *E*max (or *E*/*D* ≤ *η*max), and pick the one minimizing the normalized scalarization. This makes the weight choice a function of policy rather than an arbitrary interior point.

##### Step 5

*Sensitivity and robustness*.

We assess the stability of the selected weight(s) under parametric uncertainty:

Fuel-law parameters vary *ρ*0, *ρ*1 by ± 10% and ± 20% (independently and jointly). Further, emission factor varies *γ* by ± 10% and the operational variability, re-run with perturbed service times (± 5 min) and demand multipliers (± 10%) to emulate daily fluctuations.

For each perturbation set, we recomputed the trade-off curve, the knee, and the best policy-feasible weights. If the selected weight shifts, we report a weight interval ($\:{w}_{2}^{-}$,$\:{w}_{2}^{+}\:$) for which both $\:\widehat{F}$ and $\:\widehat{E}$ remain within 1–2% for their knee values, together with the observed variance in E/D. The stability of NV across the interval is explicitly noted.

#### Cost and emission parameters

The emission and cost parameters for the case study were set using a combination of published data and local assumptions. Referring to the Suzuki^46^ simulation data, let ρ_0_ = 1, ρ_1_ = 2. Li et al.^45^ reported that each liter of fuel consumed emits 2.32 kg of carbon dioxide. World Bank^48^ rates for effective reduction of emissions, which is $40-$80/ ton. In Pakistan, there’s no fixed cost per unit for a vehicle’s carbon emissions. So, for this study, we have considered a higher cap rate of $0.08/kg for the cost calculation, while the fuel cost is $1/ liter. These parameters were used to compute the fuel cost, carbon cost, and total operating cost in both the current and optimized routing scenarios.

#### Implementation details

We sweep the weight grid in ascending *w*2 with a resolution of 0.05 and refine to 0.025 in a narrow band around the knee; at each weight we run multiple independent trials with distinct pseudo-random seeds, recording the best solution and summarizing dispersion across runs. To accelerate convergence without biasing results, each weight is warm-started from the best solution at the previous weight, while the knee is also validated from a cold start. The evaluator is identical across all runs and scenarios and recomputed loads, waiting, and arc fuel/CO_2_ along affected suffixes after every candidate move; a move is accepted only if it improves the augmented objective and preserves feasibility. Neighborhood scheduling (intra-route before inter-route, with adaptive switching) and penalty updates are fixed across the study; no parameter is tuned per instance or per scenario. Each trial terminates at a strict local minimum that remains stable after one full penalty-update cycle or when a fixed move-evaluation budget is reached; the budget is held constant across weights and scenarios to ensure fairness.

## Results

This section consolidates the empirical evidence for the proposed E-CVRPTW and the HGLS solution strategy. It first establishes the reconstructed baseline operation for Zone D to quantify current cost and emission performance, then reports the optimized routing outcomes under the same operational constraints, and finally evaluates compliance under fleet-level environmental policy scenarios using the carbon budget and emission-intensity cap.

### Ad-hoc operation: current performance and implications

The current, non-optimized service day for Zone D of WSSP was analyzed to establish a reference point. The operation comprised 36 routes using 6 m^3^ Suzuki compactors, with route distances, fuel consumption, and CO_2_ emissions recorded for each trip. Emissions were computed from fuel usage via the emission factor γ = total CO_2_ ÷ total fuel. As shown in Table 5, the ad-hoc operation covered 708.0 km, consumed 118.0 L of diesel, and emitted 273.77 kg CO_2_, yielding an emission intensity of 0.3867 kg/km. The corresponding operational cost, comprising both fuel and carbon costs, was 133.99 USD. This baseline highlights systemic inefficiencies: excess mileage inflates fuel costs, and suboptimal routing exacerbates emissions, undermining cost-efficiency and sustainability objectives.

**Table 5:** Ad-hoc operational performance for zone D.

Run ID	Fleet config.	NV	Total distance (km)	Total fuel (L)	Total CO_2_ (kg)	Emission intensity (kg/km)	Fuel cost (USD)	Carbon cost (USD)	Total cost (USD)
Case Run A	Zone D: 6 m3 compactors	36	708	118	273.77	0.3867	112.08	21.87	133.99

The per-route structure analysis is shown in Table 7, which explains where improvements can be made. On this plan, distances are moderate, and fuel closely tracks distance across routes; route-level emission intensities are nearly constant, consistent with a stable γ. This pattern indicates that both a reduction in kilometers (via better geometry/sequence) and a reduction in fuel per kilometer (via smoother operation or vehicle efficiency) will convert almost one-for-one into CO_2_ and cost gains.

### HGLS optimized routing: efficiency gains

The Hybrid Guided Local Search (HGLS) algorithm is applied to the same dataset, minimizing route distances under operational constraints. Table 6 shows that optimization reduced total distance to 650.43 km (− 8.12%), fuel consumption to 107.28 L (− 9.08%), and CO_2_ emissions to 243.68 kg (− 11.0%). Emission intensity fell modestly to 0.3748 kg/km, while total costs dropped to 122.24 USD (− 8.73%).

**Table 6:** HGLS-optimized operational performance for zone D.

Run ID	Fleet config	NV	Total distance (km)	Total fuel (L)	Total CO_2_ (kg)	Emission intensity (kg/km)	Fuel cost (USD)	Carbon cost (USD)	Total cost (USD)
HGLS Run B	Zone D: 6 m^3^ compactors	36	650.43	107.28	243.68	0.3748	101.84	20.40	122.24

Table 7 reports the reconstructed status-quo (ad-hoc) route plan for the service day in Zone D. Each route’s distance, vehicle load, fuel use, CO_2_ emissions, and associated fuel and carbon costs are detailed, along with total operating cost and emission intensity. The fleet of 36 compactors covers 708 km, consuming 118.0 L of fuel and emitting 273.77 kg of CO_2_, yielding an average emission intensity of 0.3867 kg CO_2_/km. These figures represent the operational baseline prior to optimization and serve as the reference point for evaluating the proposed E-CVRPTW solutions.

The reconstructed status-quo routing plan represents a reconstruction of the actual operational practice followed during the relief operation. It was derived from available operational records, vehicle assignment information, and reported service sequences, and reflects the original planning logic without applying any optimization or improvement heuristic. This reconstructed plan therefore serves as a realistic baseline for comparison with the proposed optimized routing results. Table 8 presents the optimized routing plan obtained by solving the E-CVRPTW using the proposed Hybrid Guided Local Search (HGLS) without policy constraints. Compared to the ad-hoc baseline in Table 7, the optimized plan reduces total distance, fuel consumption, and CO_2_ emissions across all 36 routes, with the fleet achieving a lower overall emission intensity (average 0.3763 kg CO_2_/km). These reductions translate into measurable savings in both fuel and carbon costs while fully meeting time-window and capacity constraints. This optimized scenario demonstrates the algorithm’s capability to deliver environmental and economic improvements under unconstrained conditions, providing a basis for further evaluation in policy-constrained scenarios.

**Table 7:** Ad-hoc routes cost assessment.

Route	Distance (km)	Load (m^3^)	Fuel (L)	C_O2_ (kg)	Fuel cost	Carbon cost	Total cost ($)	Emission intensity (kg/km)	Route	Distance (km)	Load (m3)	Fuel (L)	C_O2_ (kg)	Fuel cost	Carbon cost	Total cost ($)	Emission Intensity (kg/km)
0	26.5	6	4.42	10.25	4.2	0.82	5.02	0.3868	18	19.23	6	3.21	7.44	3.04	0.59	3.64	0.3869
1	27.4	5.8	4.57	10.59	4.34	0.85	5.19	0.3865	19	25.45	5.5	4.24	9.84	4.03	0.79	4.82	0.3866
2	21.7	6	3.62	8.39	3.44	0.67	4.11	0.3866	20	18.2	6	3.03	7.04	2.88	0.56	3.44	0.3868
3	24.3	6	4.05	9.4	3.85	0.75	4.6	0.3868	21	13.88	5.7	2.31	5.37	2.2	0.43	2.63	0.3869
4	25.3	5.7	4.22	9.78	4.01	0.78	4.79	0.3866	22	18.68	6	3.11	7.22	2.96	0.58	3.54	0.3865
5	19.23	6	3.21	7.44	3.04	0.59	3.64	0.3869	23	11.75	5.8	1.96	4.54	1.86	0.36	2.22	0.3864
6	21.3	6	3.55	8.24	3.37	0.66	4.03	0.3869	24	12.75	6	2.13	4.93	2.02	0.39	2.41	0.3867
7	24.6	5.5	4.1	9.51	3.9	0.76	4.66	0.3866	25	18.55	5.4	3.09	7.17	2.94	0.57	3.51	0.3865
8	19.5	5.55	3.25	7.54	3.09	0.6	3.69	0.3867	26	16.45	6	2.74	6.36	2.6	0.51	3.11	0.3866
9	19.4	6	3.23	7.5	3.07	0.6	3.67	0.3866	27	18.25	5	3.04	7.06	2.89	0.56	3.45	0.3868
10	18.5	5.3	3.08	7.15	2.93	0.57	3.5	0.3865	28	15.6	4.8	2.6	6.03	2.47	0.48	2.95	0.3865
11	17.3	6	2.88	6.69	2.74	0.54	3.27	0.3867	29	16.76	6	2.79	6.48	2.65	0.52	3.17	0.3866
12	19.4	5.3	3.23	7.5	3.07	0.6	3.67	0.3866	30	24.85	6	4.14	9.61	3.93	0.77	4.7	0.3867
13	25.2	5.2	4.2	9.74	3.99	0.78	4.77	0.3865	31	14.55	6	2.43	5.63	2.3	0.45	2.75	0.3869
14	22.7	6	3.78	8.78	3.59	0.7	4.3	0.3868	32	18.65	6	3.11	7.21	2.95	0.58	3.53	0.3866
15	25.85	6	4.31	10	4.09	0.8	4.89	0.3868	33	18.85	5.5	3.14	7.29	2.98	0.58	3.57	0.3867
16	19.42	4.9	3.24	7.51	3.07	0.6	3.68	0.3867	34	16.55	6	2.76	6.4	2.62	0.51	3.13	0.3867
17	18.55	6	3.09	7.17	2.94	0.57	3.51	0.3865	35	12.85	5	2.14	4.97	2.03	0.4	2.43	0.3868

**Table 8:** Optimized routes cost assessment.

Route	Distance (km)	Load (m3)	Fuel (L)	C_O2_ (kg)	Fuel cost	Carbon cost	Total cost ($)	Emission intensity (kg/km)	Route	Distance (km)	Load (m3)	Fuel (L)	C_O2_ (kg)	Fuel Cost	Carbon Cost	Total Cost ($)	Emission Intensity (kg/km)
0	23.658	6	3.802	8.821	3.61	0.7	4.31	0.3729	18	17.603	6	2.886	6.696	2.74	0.53	3.27	0.3804
1	24.775	5.8	4.001	9.283	3.8	0.74	4.54	0.3747	19	23.169	5.5	3.774	8.756	3.58	0.7	4.28	0.3779
2	19.387	6	3.125	7.25	2.97	0.58	3.55	0.374	20	16.684	6	2.693	6.248	2.56	0.5	3.06	0.3745
3	22.497	6	3.692	8.566	3.51	0.68	4.19	0.3808	21	12.272	5.7	2.004	4.649	1.9	0.37	2.27	0.3788
4	21.742	5.7	3.468	8.046	3.29	0.64	3.93	0.3701	22	17.48	6	2.844	6.598	2.7	0.53	3.23	0.3775
5	17.94	6	2.964	6.877	2.82	0.55	3.37	0.3833	23	10.534	5.8	1.692	3.926	1.61	0.31	1.92	0.3727
6	18.672	6	3.004	6.97	2.85	0.56	3.41	0.3733	24	11.69	6	1.931	4.48	1.83	0.36	2.19	0.3832
7	22.592	5.5	3.61	8.376	3.43	0.67	4.1	0.3708	25	16.882	5.4	2.738	6.352	2.6	0.51	3.11	0.3763
8	17.755	5.55	2.909	6.749	2.76	0.54	3.3	0.3801	26	14.729	6	2.382	5.526	2.26	0.44	2.7	0.3752
9	17.093	6	2.776	6.441	2.64	0.51	3.15	0.3768	27	16.839	5	2.723	6.318	2.59	0.5	3.09	0.3752
10	16.02	5.3	2.581	5.988	2.45	0.48	2.93	0.3738	28	14.225	4.8	2.336	5.42	2.22	0.43	2.65	0.381
11	16.223	6	2.656	6.162	2.52	0.49	3.01	0.3798	29	15.164	6	2.486	5.768	2.36	0.46	2.82	0.3804
12	17.365	5.3	2.849	6.61	2.71	0.53	3.24	0.3807	30	23.021	6	3.733	8.661	3.55	0.69	4.24	0.3762
13	22.688	5.2	3.68	8.538	3.5	0.68	4.18	0.3763	31	13.337	6	2.169	5.032	2.06	0.4	2.46	0.3773
14	21.231	6	3.491	8.099	3.32	0.65	3.97	0.3815	32	16.754	6	2.714	6.297	2.58	0.5	3.08	0.3759
15	23.017	6	3.716	8.621	3.53	0.69	4.22	0.3745	33	17.855	5.5	2.909	6.749	2.76	0.54	3.3	0.378
16	18.09	4.9	2.975	6.902	2.83	0.55	3.38	0.3815	34	15.679	6	2.579	5.983	2.45	0.48	2.93	0.3816
17	16.79	6	2.691	6.243	2.56	0.5	3.06	0.3718	35	11.759	5	1.907	4.424	1.81	0.35	2.16	0.3762

**Table 9:** Comparison of adhoc vs HGLS (distance, fuel, and emissions).

Route	Distance adhoc	Fuel adhoc	Emission intensity adhoc	Distance HGLS	Fuel HGLS	Emission intensity HGLS	Distance % change	Fuel % change	Emission intensity % change
0	26.5	4.42	0.3868	23.658	3.802	0.3729	− 10.7245	− 13.9819	− 3.59359
1	27.4	4.57	0.3865	24.775	4.001	0.3747	− 9.58029	− 12.4508	− 3.05304
2	21.7	3.62	0.3866	19.387	3.125	0.374	− 10.659	− 13.674	− 3.25918
3	24.3	4.05	0.3868	22.497	3.692	0.3808	− 7.41975	− 8.83951	− 1.55119
4	25.3	4.22	0.3866	21.742	3.468	0.3701	− 14.0632	− 17.8199	− 4.26798
5	19.23	3.21	0.3869	17.94	2.964	0.3833	− 6.70827	− 7.66355	− 0.93047
6	21.3	3.55	0.3869	18.672	3.004	0.3733	− 12.338	− 15.3803	− 3.51512
7	24.6	4.1	0.3866	22.592	3.61	0.3708	− 8.1626	− 11.9512	− 4.08691
8	19.5	3.25	0.3867	17.755	2.909	0.3801	− 8.94872	− 10.4923	− 1.70675
9	19.4	3.23	0.3866	17.093	2.776	0.3768	− 11.8918	− 14.0557	− 2.53492
10	18.5	3.08	0.3865	16.02	2.581	0.3738	− 13.4054	− 16.2013	− 3.2859
11	17.3	2.88	0.3867	16.223	2.656	0.3798	− 6.22543	− 7.77778	− 1.78433
12	19.4	3.23	0.3866	17.365	2.849	0.3807	− 10.4897	− 11.7957	− 1.52613
13	25.2	4.2	0.3865	22.688	3.68	0.3763	− 9.96825	− 12.381	− 2.63907
14	22.7	3.78	0.3868	21.231	3.491	0.3815	− 6.47137	− 7.6455	− 1.37022
15	25.85	4.31	0.3868	23.017	3.716	0.3745	− 10.9594	− 13.7819	− 3.17994
16	19.42	3.24	0.3867	18.09	2.975	0.3815	− 6.84861	− 8.17901	− 1.34471
17	18.55	3.09	0.3865	16.79	2.691	0.3718	− 9.48787	− 12.9126	− 3.80336
18	19.23	3.21	0.3869	17.603	2.886	0.3804	− 8.46074	− 10.0935	− 1.68002
19	25.45	4.24	0.3866	23.169	3.774	0.3779	− 8.96267	− 10.9906	− 2.25039
20	18.2	3.03	0.3868	16.684	2.693	0.3745	− 8.32967	− 11.1221	− 3.17994
21	13.88	2.31	0.3869	12.272	2.004	0.3788	− 11.585	− 13.2468	− 2.09356
22	18.68	3.11	0.3865	17.48	2.844	0.3775	− 6.42398	− 8.55305	− 2.32859
23	11.75	1.96	0.3864	10.534	1.692	0.3727	− 10.3489	− 13.6735	− 3.54555
24	12.75	2.13	0.3867	11.69	1.931	0.3832	− 8.31373	− 9.34272	− 0.90509
25	18.55	3.09	0.3865	16.882	2.738	0.3763	− 8.99191	− 11.3916	− 2.63907
26	16.45	2.74	0.3866	14.729	2.382	0.3752	− 10.462	− 13.0657	− 2.94878
27	18.25	3.04	0.3868	16.839	2.723	0.3752	− 7.73151	− 10.4276	− 2.99897
28	15.6	2.6	0.3865	14.225	2.336	0.381	− 8.8141	− 10.1538	− 1.42303
29	16.76	2.79	0.3866	15.164	2.486	0.3804	− 9.52267	− 10.8961	− 1.60372
30	24.85	4.14	0.3867	23.021	3.733	0.3762	− 7.36016	− 9.83092	− 2.71528
31	14.55	2.43	0.3869	13.337	2.169	0.3773	− 8.33677	− 10.7407	− 2.48126
32	18.65	3.11	0.3866	16.754	2.714	0.3759	− 10.1662	− 12.7331	− 2.76772
33	18.85	3.14	0.3867	17.855	2.909	0.378	− 5.27851	− 7.35669	− 2.24981
34	16.55	2.76	0.3867	15.679	2.579	0.3816	− 5.26284	− 6.55797	− 1.31885
35	12.85	2.14	0.3868	11.759	1.907	0.3762	− 8.49027	− 10.8879	− 2.74043

Table 9 provides a detailed route-by-route comparison between the reconstructed ad hoc baseline (Table 7) and the optimized HGLS solution (Table 8) for distance, fuel consumption, and emission intensity. The percentage change columns quantify the relative improvement achieved for each route. Across all 36 routes, the HGLS solution consistently reduces distance (up to 14.06%), fuel use (up to 17.82%), and emission intensity (up to 4.27%), with average reductions of approximately 9–11% in distance and fuel, and around 2–3% in emission intensity. These results confirm the algorithm’s capacity to achieve route- level efficiency gains while meeting operational constraints, demonstrating that improvements are distributed throughout the service network rather than concentrated in only a few routes.

**Fig. 5:**
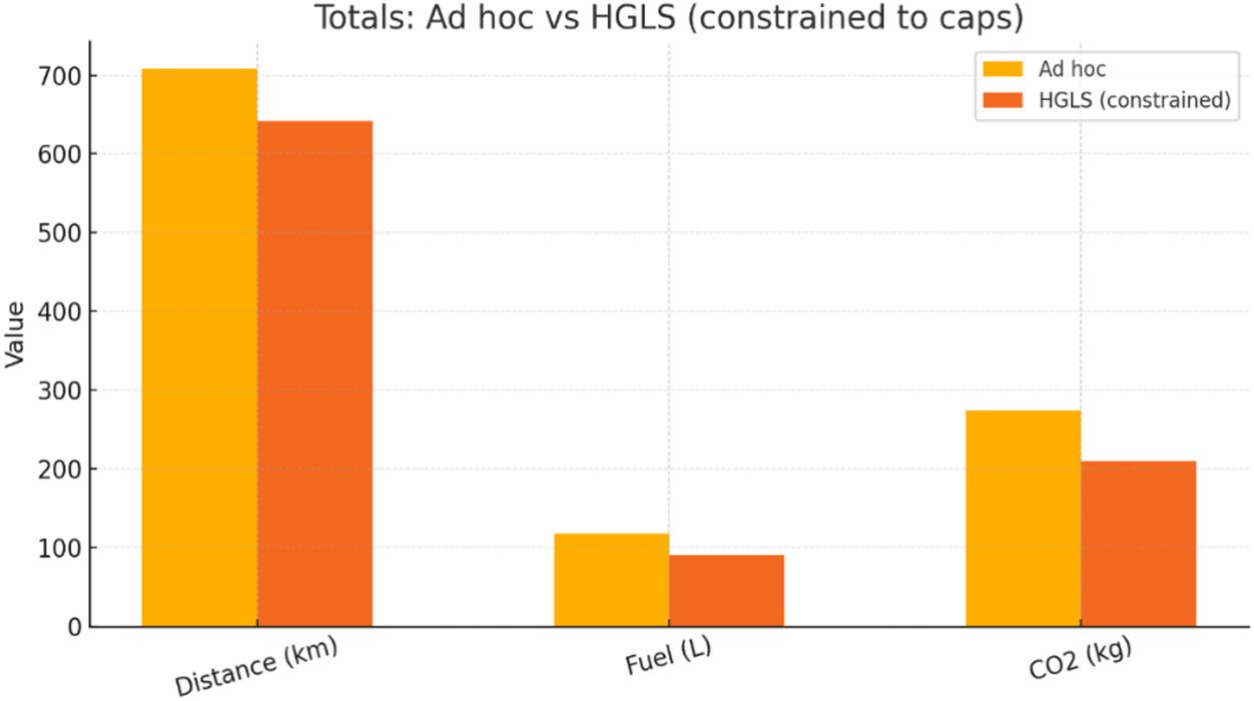
Per-route fuel and distance change (Ad-hoc vs. HGLS).

The per-route comparison (Fig. 5; Table 9) reveals substantial reductions for most routes (~ 8–12%), although high-density clusters show marginal gains due to demand concentration and limited consolidation potential. This indicates that the optimization potential is route-dependent, shaped by spatial dispersion and payload-balancing constraints.

### Scenario analysis: policy-based constraints

To evaluate alignment with sustainability objectives, six operational scenarios (S0–S5) are tested against two policy constraints: a daily carbon budget of 246.39 kg CO_2_ and an emission intensity cap of 0.3480 kg/km. These thresholds are derived from regional sustainability targets and World Bank guidance. The scenarios varied in routing strategy, fleet configuration, and operational constraints:

i. S0 Ad hoc – Baseline, non-optimized routing.
ii. S1 HGLS (constrained) – Distance-minimizing HGLS routing under emission constraints.
iii. S2 Knee – HGLS run at the knee-point of the Pareto front for cost–emission trade-off.
iv. S3 FleetSwap – Fleet composition change to 3 m^3^ mini trucks with optimized routing.
v. S4 Budget – Mini-truck fleet optimized with explicit carbon budget constraint.
vi. S5 IntensityCap – Mini-truck fleet optimized with explicit emission intensity constraint.

Table 10 presents the results for each scenario, including total distance, fuel, CO_2_ emissions, emission intensity, and compliance with the policy thresholds.

**Table 10:** Multi-scenario performance and compliance with carbon budget and emission intensity cap.

Scenario	Fleet config	NV	Total distance (km)	Total fuel (L)	Total CO_2_ (kg)	Emission intensity (kg/km)	Meets carbon budget?	Meets intensity cap?
S_0_ Ad hoc	Zone D: 6 m^3^ compactors	36	708.00	118.00	273.77	0.3867	No	No
S1 HGLS (constrained)	Zone D: 6 m^3^ compactors	36	642.07	90.48	209.92	0.3269	Yes	Yes
S2 Knee	Zone D: 6 m^3^ compactors	36	642.07	90.03	208.87	0.3253	Yes	Yes
S3 FleetSwap	Zone D: 3 m^3^ mini trucks	36	651.70	82.65	191.76	0.2942	Yes	Yes
S4 Budget	Zone D: 3 m^3^ mini trucks	36	651.70	82.65	191.76	0.2942	Yes	Yes
S5 IntensityCap	Zone D: 3 m^3^ mini trucks	36	651.70	82.65	191.76	0.2942	Yes	Yes

The expanded scenario set underscores a key insight: emission intensity reductions are not solely a function of distance minimization. Although the HGLS-optimized 6 m^3^ compactor scenarios (S1–S2) show significant improvement over the baseline (S0), they remain less efficient than the mini-truck scenarios (S3–S5). This difference reflects Peshawar’s urban realities—narrow, congested streets, minimal road expansion despite rapid vehicle growth, and frequent traffic bottlenecks—that disadvantage high-capacity compactors even under optimized routing. Peshawar is among the most polluted cities globally, with PM_2_.₅ concentrations far exceeding WHO guidelines, and vehicular emissions are a leading contributor to this pollution. In dense, stop-and-go traffic, larger compactor engines are less fuel-efficient, whereas smaller mini-trucks maneuver more effectively and maintain lower per-kilometer fuel consumption. These results highlight the complementary roles of routing optimization and fleet configuration. While algorithmic optimization (S1–S2) achieves substantial gains, deeper reductions, particularly in emission intensity, require operational strategies like load rebalancing, congestion-aware scheduling, and driver eco-training. Selective fleet electrification for low-volume or highly congested routes could yield an additional ~ 20% reduction in CO_2_ emissions without route redesign, offering both environmental and public health benefits amid Peshawar’s current air quality crisis.

## Conclusion

This study presents the Emission-Capacitated Vehicle Routing Problem with Time Windows (E-CVRPTW), which integrates a load-dependent fuel model with an emissions term and policy constraints. The objective is to optimize both fuel consumption and CO_2_ emissions while ensuring the routing and timing constraints of the VRPTW are maintained. This allows managers to make decisions based on cost, carbon emissions, or a balanced compromise by adjusting simple weight parameters. To solve this problem, the study employs a scalable HGLS, which combines a novel CFI seed with a penalty-driven Guided Local Search approach. The algorithm alternates adaptively between 2-opt/3-opt and inter-route moves, ensuring feasibility after each candidate moves and triggering neighborhood switching when further improvements stagnate.

The proposed HGLS method is evaluated against community benchmarks and demonstrates competitive performance. On clustered Solomon instances, it matches the best-known vehicle configurations, and on random and mixed sets, it reduces vehicle counts. For larger-scale instances, such as the Gehring–Homberger benchmark, the method consistently improves distance over simulated annealing by 10–14%, without increasing the number of vehicles. This demonstrates the scalability and stability of the approach. When applied to Water and Sanitation Services Peshawar (Zone D), the model incorporates real-world access rules, time windows, and service durations for 109 containerized sites. The optimization respects a carbon budget and intensity cap, ensuring compliance while optimizing fuel use and emissions. The results highlight that routing alone can drive substantial carbon reductions, particularly by eliminating unnecessary re-entries and heavy-load deadheading. Furthermore, the study demonstrates that vehicle size should align with street geometry and demand to minimize emissions. At the same time, policy instruments such as a carbon budget and an intensity cap complement each other in achieving emission targets.

Beyond quantifying fuel and emission reductions, the scenario framework (S0 to S5) offers a structured decision support tool for practitioners to evaluate operational and policy trade-offs before implementation. Each scenario represents a distinct governance posture ranging from baseline replication (S0) to cost driven optimization (S1), balanced trade-off (S2), and policy constrained strategies (S3 to S5). By comparing these scenarios, municipal planners can identify how different regulatory levers such as carbon budgets or emission intensity caps reshape route geometry, fleet utilization, and cost structure. For instance, the balanced (S2) and budget constrained (S4) solutions demonstrate that moderate emission caps can achieve compliance with minimal cost sacrifice, guiding the design of feasible carbon policies for daily operations. Similarly, the fleet swap scenario (S3) reveals when shifting to smaller or low emission vehicles yields greater marginal benefit than tightening route constraints. Hence, the E-CVRPTW framework does not prescribe a single optimal plan but provides a policy adaptive planning instrument, allowing practitioners to test multiple strategies and select those aligning with local infrastructure, resource availability, and sustainability priorities. Although the study focuses on a single day and zone, it paves the way for future research in responsive operations, real-time re-optimization, and the integration of multi-period planning and fleet strategies.

## Supplementary Information

Below is the link to the electronic supplementary material.

Supplementary Material 1

## Data Availability

The datasets used and/or analysed during the current study available from the corresponding author on reasonable request.
