# Supplementary material for: An emission-capacitated vehicle routing model for sustainable urban waste collection using hybrid guided local search
Source: Sci Rep. 2026 Feb 7;16:7691. doi: 10.1038/s41598-026-38829-5 (PMC12946196; doi:10.1038/s41598-026-38829-5)
Supplement: Supplementary file 1 — Supplementary Material 1 [file 41598_2026_38829_MOESM1_ESM.docx]

**Appendix**

Table A1. Input data of the WSSP Problem

| Point | x | y | a_i_ | b_i_ | Demand | Service Time | Point | x | y | a_i_ | b_i_ | Demand | Service Time |
| --- | --- | --- | --- | --- | --- | --- | --- | --- | --- | --- | --- | --- | --- |
| 0 | 34.045903 | 71.532091 | 8:00 | 12:00 | 2m^3^ | 46 | 56 | 34.021771 | 71.535448 | 8:00 | 16:00 | 2m^3^ | 60 |
| 1 | 34.00333 | 71.46727 | 8:00 | 13:30 | 2m^3^ | 60 | 57 | 34.039361 | 71.527272 | 8:00 | 12:00 | 2m^3^ | 49 |
| 2 | 33.999504 | 71.469066 | 8:00 | 16:00 | 2m^3^ | 51 | 58 | 34.031501 | 71.531981 | 8:00 | 13:30 | 2m^3^ | 46 |
| 3 | 34.00073 | 71.468739 | 8:00 | 12:00 | 2m^3^ | 60 | 59 | 34.025736 | 71.535138 | 8:00 | 16:00 | 2m^3^ | 56 |
| 4 | 34.00073 | 71.468731 | 8:00 | 13:30 | 2m^3^ | 56 | 60 | 34.019218 | 71.537193 | 8:00 | 12:00 | 2m^3^ | 56 |
| 5 | 33.99954 | 71.47014 | 8:00 | 16:00 | 2m^3^ | 51 | 61 | 34.026788 | 71.534556 | 8:00 | 13:30 | 2m3 | 45 |
| 6 | 34.00497 | 71.49059 | 8:00 | 12:00 | 2m^3^ | 47 | 62 | 34.039541 | 71.524646 | 8:00 | 16:00 | 2m^3^ | 52 |
| 7 | 34.00164 | 71.49126 | 8:00 | 13:30 | 2m^3^ | 51 | 63 | 34.030528 | 71.528913 | 8:00 | 12:00 | 2m^3^ | 54 |
| 8 | 34.003 | 71.4929 | 8:00 | 16:00 | 2m^3^ | 46 | 64 | 34.003504 | 71.467207 | 8:00 | 13:30 | 2m^3^ | 51 |
| 9 | 34.00488 | 71.49536 | 8:00 | 12:00 | 2m^3^ | 60 | 65 | 34.014716 | 71.49068 | 8:00 | 16:00 | 2m^3^ | 59 |
| 10 | 34.00484 | 71.4966 | 8:00 | 13:30 | 2m^3^ | 53 | 66 | 34.01838 | 71.536411 | 8:00 | 12:00 | 2m^3^ | 53 |
| 11 | 34.00243 | 71.49494 | 8:00 | 16:00 | 2m^3^ | 56 | 67 | 34.002441 | 71.500136 | 8:00 | 13:30 | 2m^3^ | 51 |
| 12 | 34.0099 | 71.51991 | 8:00 | 12:00 | 2m^3^ | 59 | 68 | 34.009033 | 71.512861 | 8:00 | 16:00 | 2m^3^ | 58 |
| 13 | 34.010696 | 71.520983 | 8:00 | 13:30 | 2m^3^ | 57 | 69 | 34.025491 | 71.52435 | 8:00 | 12:00 | 0.8m^3^ | 44 |
| 14 | 34.013037 | 71.525471 | 8:00 | 16:00 | 2m^3^ | 60 | 70 | 34.017475 | 71.538929 | 8:00 | 13:30 | 0.8m^3^ | 36 |
| 15 | 34.008249 | 71.519407 | 8:00 | 12:00 | 2m^3^ | 46 | 71 | 34.023158 | 71.524134 | 8:00 | 16:00 | 0.8m^3^ | 43 |
| 16 | 34.013067 | 71.504997 | 8:00 | 13:30 | 2m^3^ | 57 | 72 | 34.024893 | 71.518937 | 8:00 | 12:00 | 0.8m^3^ | 45 |
| 17 | 34.009598 | 71.510711 | 8:00 | 16:00 | 2m^3^ | 48 | 73 | 34.016099 | 71.49382 | 8:00 | 13:30 | 0.8m^3^ | 43 |
| 18 | 34.008798 | 71.515354 | 8:00 | 12:00 | 2m^3^ | 58 | 74 | 34.014963 | 71.507461 | 8:00 | 16:00 | 0.8m^3^ | 45 |
| 19 | 34.01092 | 71.51756 | 8:00 | 13:30 | 2m^3^ | 52 | 75 | 34.014903 | 71.496563 | 8:00 | 12:00 | 0.8m^3^ | 35 |
| 20 | 34.01575 | 71.52402 | 8:00 | 16:00 | 2m^3^ | 45 | 76 | 34.027705 | 71.49548 | 8:00 | 13:30 | 0.8m^3^ | 41 |
| 21 | 34.009 | 71.51865 | 8:00 | 12:00 | 2m^3^ | 52 | 77 | 34.003775 | 71.5054464 | 8:00 | 16:00 | 0.8m^3^ | 43 |
| 22 | 34.01314 | 71.5137262 | 8:00 | 13:30 | 2m^3^ | 58 | 78 | 34.013168 | 71.487469 | 8:00 | 12:00 | 0.8m^3^ | 41 |
| 23 | 34.01102 | 71.50221 | 8:00 | 16:00 | 2m^3^ | 49 | 79 | 34.027645 | 71.495552 | 8:00 | 13:30 | 0.8m^3^ | 39 |
| 24 | 34.01001 | 71.508988 | 8:00 | 12:00 | 2m^3^ | 60 | 80 | 34.019988 | 71.459826 | 8:00 | 16:00 | 0.8m^3^ | 41 |
| 25 | 34.01286 | 71.503007 | 8:00 | 13:30 | 2m^3^ | 48 | 81 | 34.003057 | 71.481551 | 8:00 | 12:00 | 0.8m^3^ | 44 |
| 26 | 34.017539 | 71.498173 | 8:00 | 16:00 | 2m^3^ | 48 | 82 | 34.004313 | 71.471807 | 8:00 | 13:30 | 0.8m^3^ | 42 |
| 27 | 34.005427 | 71.50145 | 8:00 | 12:00 | 2m^3^ | 47 | 83 | 34.013766 | 71.466249 | 8:00 | 16:00 | 0.8m^3^ | 42 |
| 28 | 34.015735 | 71.493051 | 8:00 | 13:30 | 2m^3^ | 49 | 84 | 34.003595 | 71.48386 | 8:00 | 12:00 | 0.8m^3^ | 42 |
| 29 | 34.01176 | 71.5022 | 8:00 | 16:00 | 2m^3^ | 53 | 85 | 34.036244 | 71.490414 | 8:00 | 13:30 | 0.8m^3^ | 39 |
| 30 | 34.00565 | 71.49718 | 8:00 | 12:00 | 2m^3^ | 54 | 86 | 34.026285 | 71.474107 | 8:00 | 16:00 | 0.8m^3^ | 39 |
| 31 | 34.00373 | 71.50043 | 8:00 | 13:30 | 2m^3^ | 60 | 87 | 34.003519 | 71.46827 | 8:00 | 12:00 | 0.8m^3^ | 36 |
| 32 | 34.01936 | 71.49216 | 8:00 | 16:00 | 2m^3^ | 53 | 88 | 34.0011 | 71.492389 | 8:00 | 13:30 | 0.8m^3^ | 39 |
| 33 | 34.023327 | 71.492933 | 8:00 | 12:00 | 2m^3^ | 60 | 89 | 34.03226 | 71.462777 | 8:00 | 16:00 | 0.8m^3^ | 39 |
| 34 | 34.023323 | 71.485849 | 8:00 | 13:30 | 2m^3^ | 60 | 90 | 34.029771 | 71.472905 | 8:00 | 12:00 | 0.8m^3^ | 45 |
| 35 | 34.031121 | 71.488446 | 8:00 | 16:00 | 2m^3^ | 45 | 91 | 34.020879 | 71.470502 | 8:00 | 13:30 | 0.8m^3^ | 44 |
| 36 | 34.019097 | 71.492207 | 8:00 | 12:00 | 2m^3^ | 47 | 92 | 34.026351 | 71.443279 | 8:00 | 16:00 | 0.8m^3^ | 45 |
| 37 | 34.034403 | 71.486495 | 8:00 | 13:30 | 2m^3^ | 46 | 93 | 33.996484 | 71.44777 | 8:00 | 12:00 | 0.8m^3^ | 41 |
| 38 | 34.0313 | 71.48849 | 8:00 | 16:00 | 2m^3^ | 52 | 94 | 33.995638 | 71.47043 | 8:00 | 13:30 | 0.8m^3^ | 38 |
| 39 | 34.00873 | 71.4615 | 8:00 | 12:00 | 2m^3^ | 53 | 95 | 34.004015 | 71.467776 | 8:00 | 16:00 | 0.8m^3^ | 38 |
| 40 | 34.008951 | 71.518679 | 8:00 | 13:30 | 2m^3^ | 60 | 96 | 34.013322 | 71.478902 | 8:00 | 12:00 | 0.8m^3^ | 38 |
| 41 | 34.001769 | 71.422714 | 8:00 | 16:00 | 2m^3^ | 50 | 97 | 33.990814 | 71.489694 | 8:00 | 13:30 | 0.8m^3^ | 38 |
| 42 | 34.001208 | 71.420222 | 8:00 | 12:00 | 2m^3^ | 53 | 98 | 33.989908 | 71.485446 | 8:00 | 16:00 | 0.8m^3^ | 43 |
| 43 | 34.003298 | 71.467093 | 8:00 | 13:30 | 2m^3^ | 52 | 99 | 33.981755 | 71.493457 | 8:00 | 12:00 | 0.8m^3^ | 35 |
| 44 | 34.008163 | 71.46318 | 8:00 | 16:00 | 2m^3^ | 57 | 100 | 33.997355 | 71.501954 | 8:00 | 13:30 | 0.8m^3^ | 40 |
| 45 | 34.0132 | 71.466378 | 8:00 | 12:00 | 2m^3^ | 54 | 101 | 33.989304 | 71.456314 | 8:00 | 16:00 | 0.8m^3^ | 42 |
| 46 | 34.014361 | 71.461538 | 8:00 | 13:30 | 2m^3^ | 58 | 102 | 33.991719 | 71.510329 | 8:00 | 12:00 | 0.8m^3^ | 45 |
| 47 | 34.021828 | 71.458935 | 8:00 | 16:00 | 2m^3^ | 50 | 103 | 33.982158 | 71.511907 | 8:00 | 13:30 | 0.8m^3^ | 45 |
| 48 | 34.006391 | 71.462707 | 8:00 | 12:00 | 2m^3^ | 52 | 104 | 34.014461 | 71.502075 | 8:00 | 16:00 | 0.8m^3^ | 35 |
| 49 | 34.031861 | 71.470646 | 8:00 | 13:30 | 2m^3^ | 59 | 105 | 33.994437 | 71.522225 | 8:00 | 12:00 | 0.8m^3^ | 43 |
| 50 | 34.012637 | 71.43433763 | 8:00 | 16:00 | 2m^3^ | 46 | 106 | 33.999066 | 71.500133 | 8:00 | 13:30 | 0.8m^3^ | 44 |
| 51 | 34.013056 | 71.435566 | 8:00 | 12:00 | 2m^3^ | 52 | 107 | 34.002286 | 71.48180465 | 8:00 | 16:00 | 0.8m^3^ | 39 |
| 52 | 34.011265 | 71.434363 | 8:00 | 13:30 | 2m^3^ | 49 | 108 | 34.00772 | 71.483139 | 8:00 | 12:00 | 0.8m^3^ | 37 |
| 53 | 34.010374 | 71.447001 | 8:00 | 16:00 | 2m^3^ | 46 | 109 | 34.045903 | 71.532091 | 8:00 | 13:30 | 0.8m^3^ | 45 |
| 54 | 34.013898 | 71.433799 | 8:00 | 12:00 | 2m^3^ | 50 | 110 | 34.00333 | 71.46727 | 8:00 | 16:00 | 0.8m^3^ | 40 |
| 55 | 34.010068 | 71.432005 | 8:00 | 13:30 | 2m^3^ | 53 |  |  |  |  |  |  |  |
